# Genome-wide identification and expression analysis of dirigent-jacalin genes from plant chimeric lectins in Moso bamboo (*Phyllostachys edulis*)

**DOI:** 10.1371/journal.pone.0248318

**Published:** 2021-03-16

**Authors:** Ruifang Ma, Bin Huang, Jialu Chen, Zhinuo Huang, Peiyao Yu, Shiyu Ruan, Zhijun Zhang

**Affiliations:** 1 State Key Laboratory of Subtropical Forest Cultivation, Zhejiang A&F University, Lin’an, Hangzhou, Zhejiang, China; 2 School of Forestry and Biotechnology, Zhejiang A&F University, Lin’an, Hangzhou, Zhejiang, China; University of Hyderabad, INDIA

## Abstract

Dirigent-jacalin (D-J) genes belong to the plant chimeric lectin family, and play vital roles in plant growth and resistance to abiotic and biotic stresses. To explore the functions of the D-J family in the growth and development of Moso bamboo (*Phyllostachys edulis)*, their physicochemical properties, phylogenetic relationships, gene and protein structures, and expression patterns were analyzed in detail. Four putative *PeD-J* genes were identified in the Moso bamboo genome, and microsynteny and phylogenetic analyses indicated that they represent a new branch in the evolution of plant lectins. PeD-J proteins were found to be composed of a dirigent domain and a jacalin-related lectin domain, each of which contained two different motifs. Multiple sequence alignment and homologous modeling analysis indicated that the three-dimensional structure of the PeD-J proteins was significantly different compared to other plant lectins, primarily due to the tandem dirigent and jacalin domains. We surveyed the upstream putative promoter regions of the *PeD-Js* and found that they mainly contained *cis*-acting elements related to hormone and abiotic stress response. An analysis of the expression patterns of root, leaf, rhizome and panicle revealed that four *PeD-J* genes were highly expressed in the panicle, indicating that they may be required during the formation and development of several different tissue types in Moso bamboo. Moreover, *PeD-J* genes were shown to be involved in the rapid growth and development of bamboo shoots. Quantitative Real-time PCR (qRT PCR) assays further verified that D-J family genes were responsive to hormones and stresses. The results of this study will help to elucidate the biological functions of *PeD-Js* during bamboo growth, development and stress response.

## Introduction

Moso bamboo (*Phyllostachys edulis*) is a major species of woody bamboo that is widely cultivated in tropical and subtropical regions [1]. It belongs to the grass family Poaceae, subfamily Bambusoideae, and has high ecological, economic and cultural value [2]. Bamboo timber is also utilized in furniture, paper and charcoal making [3]. Additionally, Moso bamboo shoots are used as a food source in many regions [4]. Moso bamboo has an active growth period from March to May, and its shoots can grow to a height of about 500 cm within a week [5]. For its rapid growth, shoots can reach a height of more than 20 m within 2 months [2]. Due to its high growth rate, Moso bamboo needs to be able to respond to biotic and abiotic stresses in a rapid manner. A better understanding of Moso bamboo’s genome and transcriptome may also yield new genetic resources for the improvement of other species [6].

Plants have evolved adaptations to many different adverse external environments, such as high salinity soil, drought, heat, cold, pests and diseases [7–10]. As a result, plants have developed numerous sophisticated physiological, cellular and molecular mechanisms, such as disease resistance genes, stress response transcription factors, lectins and a host of other mechanisms to adapt to adverse conditions [11–15]. The study of these genes in bamboo will deepen the current understanding of the evolutionary and functional mechanisms of plant adaptation to the environment, which will be beneficial to the improvement of crop genetics.

Lectins, also known as hemagglutinins, are a diverse group of non-immunogenic glycoproteins that exist in microorganisms, animals and plants [16, 17]. They contain at least one non-catalytic domain that reversibly binds to a specific glycan structure [18]. Plant-derived lectins are a class of proteins with specific glycan-binding activity, whose primary function is to participate in the resistance of host cells against abiotic and biotic stresses [19–21]. In the 1880s, Stillmarck identified the first plant lectin: a red blood cell clotting factor found in castor seed extract [22, 23]. Many plant lectins are unaffected by the environment and are expressed in a constitutive manner within various nutrient storage tissues, such as seeds, bark, bulbs and rhizomes [24]. Most of these constitutively expressed lectins are synthesized with signal peptides and enter secretory pathways [16]. Several recent studies have shown that the expression of some lectin genes can be induced under certain stresses, such as intense light, drought and cold [25–27]. Lectin genes not only respond to biotic and abiotic stresses but are also involved in the defense pathway of several plant hormones, such as SA, ABA, and MeJA. Barley *Lem2* expression was strongly up-regulated by SA and its functional analogue 2,6-dichloroisonicotinic acid showed induction at 4 h and was down-regulated by drought and ABA treatment, but not in response to MeJA [28]; *Arabidopsis At5g28520* was up-regulated strongly by ABA induction and responded at seedling stage to ABA signaling pathway induction [29]. *Hfr-1* was induced by SA and *WCI-1* was induced by SA and JA [25]. In contrast to the abundant constitutive lectins, inducible lectins are typically localized to the plant nucleus and cytoplasm, and have low to moderate expression in roots, leaves and flowers. Despite their low abundance, these lectins may also interact with sugars inside plant cells or in the plant cell wall to initiate a series of cell-to-cell signaling pathways [16, 30].

JRL (Jacalin-Related Lectin) is a family of plant lectins, which can be classified into two categories based on sugar-binding specificity. Class one JRLs are the galactose-specific jacalin lectins (galactose-specific lectins, gJRLs), which includes jacalin agglutinin of panasa (*Artocarpus heterophyllus*) [31], MPA agglutinin of ossessan orange (*Maclura pomifera*) [32] and black bilberry agglutinin (*Morus nigra*) [33]. This type of lectin has peptide chains and a signal peptide at the N-terminus, which is removed during post-translational transmembrane transport, and are mostly localized to the vesicles of cells. The other type of JRLs are mannose- or glucose-specific jacalin lectins (mJRLs). Most of the JRLs discovered so far are mJRLs, which lack a signal peptide and are usually located in the cytoplasm or nucleus [28, 34–36]. Subcellular localization prediction of JRLs in rice, *Arabidopsis* and wheat indicates that most are likely localized to the nucleus or cytoplasm [37].

Based on their overall structural characteristics, plant lectins are divided into four categories, including partial lectins, total lectins, super lectins and chimeric lectins [24]. Chimeric lectins have at least one jacalin domain containing glycan binding sites. Most jacalin proteins (JRLs) contain only a jacalin domain, but there are some N- or C-terminal chimeric JRLs that contain other domains such as dirigent, NB-ARC, F-Box, GNA and PAG domains [38]. In Gramineae (maize, sorghum, rice, wheat, sugarcane and others), a unique type of chimeric dirigent-jacalin (D-J) lectin is common [39–45]. These genes play essential roles in plants, responding to biotic and abiotic stresses, as well as regulating plant growth and development [46, 47]. Co-evolutionary analysis has shown that there is a significant linkage between the dirigent and jacalin domains in the D-J family. However, because the D-J genes contain both dirigent and jacalin domains, they appear in both the JRL and DIR families. Thus, the exact categorization of this family is still uncertain.

The dirigent domain is a common sequence feature of DIR family proteins, which primarily function in the biosynthesis of cell wall lignin and lignan in response to pests, diseases and abiotic stresses [48, 49]. DIR proteins typically contain several glycosylation sites, and a signal peptide sequence associated with the secretory pathway at the N-terminus. DIR proteins also contain eight reverse parallel chains forming a β-barrel structure, with an inner hydrophobic substrate-binding cavity [50]. The recent identification of a new dirigent domain suggests that such chimeric D-J lectins may be more functionally diverse than previously thought [51, 52]. Studies have shown that DIR genes are involved in disease resistance responses and may affect the sugar-binding specificity of the entire lectin protein [41, 51]. In sugarcane (*Saccharum officinarum*), JRL domains affiliated with a dirigent domain (Dirigent-Jacalin or D-J) have been shown to play a part in drought tolerance [53].

Chimeric dimeric-jacalin (D-J) lectins have special structural properties and biological properties compared to other species of plant lectin family. Andrade et al. [53] described the role that the JRL domain plays in conjunction with the dirigent domain (dirigent-jacalin, or DJ) in drought tolerance, thus demonstrating the role of chimeric lectin in abiotic stress. Thus far, genome-wide analysis of SBP-like [53], ARF [54], MADS-box [55], UBP [56], MYB [57] and other gene families has been performed in Moso bamboo, but there is little known about the occurrence of different D-J lectins within bamboo species, despite the sequencing of its genome several years ago [58]. The new assembly of the Moso bamboo genome offers an opportunity to improve our understanding of the abundance, distribution and expansion of bamboo lectins [59]. In this study, we identified four Moso bamboo chimeric D-J lectin genes and investigated the structural domains, expansion patterns and evolutionary relationship among bamboo lectins.

## Materials and methods

### Identification of D-J gene family members in Moso bamboo

To determine the presence of the D-J genes in Moso bamboo, HMMER3 [60] was used to search for the dirigent domain (PF03018) and the jacalin domain (PF01419) in the Moso bamboo genome database (ftp://parrot.genomics.cn/gigadb/pub/10.5524/100001_101000/100498/). Only hits with E-values less than or equal to 10^−10^ were chosen as candidates. Venn diagram analysis was used to identify sequences that contained both a dirigent domain and a jacalin domain (D-J genes) using online software (http://bioinformatics.psb.ugent.be/webtools/Venn/) [61]. Using the above criteria, we also screened D-J genes from the genome databases of other plant species, including barley, wheat, rice and corn, which were downloaded from Ensembl (http://www.ensembl.org/index.html) and Phytozome (https://phytozome.jgi.doe.gov/pz/portal.html). The ExPASy ProtParam (https://web.expasy.org/protparam/) [62] and SignalP 4.1 Server [63] (http://www.cbs.dtu.dk/services/SignalP-4.1/) were used to predict basic physicochemical properties and make predictions about signal peptides for each gene family member. The subcellular localization prediction software ApoplastP was used to predict the localization of PeD-J proteins (http://apoplastp.csiro.au/). The identified D-J genes were named *PeD-J01* to *PeD-J04*, according to their positions on Moso bamboo chromosomes.

### Chromosomal location and collinearity analyses

All genes of *PeD-Js*, *PeDIRs* and *PeJRLs* were mapped to the pseudomolecules using TBtools software v1.045 [64]. Gene collinearity analyses were performed using the MCScanX program [65] based on the GFF3 files of Moso bamboo. Genes that lacked placement on major chromosomes (Scaffold1—Scffold24, removing Scaffold17, Scaffold18, Scaffold19 and Scaffold20) were excluded from examination. A schematic diagram of the presumed duplications was generated with the TBtools program.

### Phylogenetic analysis

The dirigent, jacalin and D-J protein sequences of bamboo were analyzed by an intraspecific phylogenetic tree. The D-J protein sequences extracted from barley, wheat, rice, maize and bamboo were used to construct an inter-species phylogenetic tree. Geneious software was used to implement multiple alignments of amino acid sequences, and next, the neighbor-joining (NJ) method in MEGA7.0 was used to construct interspecific and intraspecific phylogenetic trees [66]. The confidence coefficient of the resulting trees were measured by 1,000 bootstraps with the pairwise deletion option.

### Gene structures, conserved domains and motifs of *PeD-Js*

Gene structures of *PeD-Js* were analyzed according to the information in the whole-genome GFF annotation file from Moso bamboo. To identify additional conserved domains of PeD-Js, the protein sequences were searched against the NCBI Conserved Domain (https://www.ncbi.nlm.nih.gov/cdd/), Simple Molecular Agriculture Research Tool (SMART) (http://smart.embl-heidelberg.de/) and EMBL-Pfam (https://pfam.xfam.org/) databases. In addition, the full-length protein sequences of PeD-Js were submitted to Multiple Expectation Maximization for Motif Elicitation (MEME) (http://meme-suite.org/tools/meme) [67] with an optimum of 6–50 residues in width and a maximum of 5 motifs. Schematic domain and motif distribution diagrams of *PeD-Js* were edited with IBS software (http://ibs.biocuckoo.org/online.php) [68].

### Homology modeling

Homology modeling was used to predict the 3D structures of PeD-J proteins based on their similarity to proteins with known structures. The appropriate homologous templates were retrieved from the PDB database (http://www.rcsb.org/). The main chain, side chain and loop region of the tertiary structures of proteins was modeled by EasyModeller software (http://softwaretopic.informer.com/easy-modeller-for-windows/). The optimum models were generated by the SAVES server (https://servicesn.mbi.ucla.edu/SAVES/). Predicted model structures were visualized and manipulated with Discovery Studio, version 2016 (BIOVIA).

### Promoter motif analysis

The 1.5-kbp upstream of the ATG start site from each *PeD-J* coding sequence was considered to be promoter region and extracted from the Moso bamboo genome. The *cis*-acting regulatory DNA motifs were predicted by PlantCARE software (http://bioinformatics.psb.ugent.be/webtools/plantcare/html/) [69] and classified to determine elements with functional relevance. The schematic representation of their promoter motif was visualized by TBtools.

### Heat map of gene expression

Several transcriptome libraries (registration numbers SRR6171235, SRR6171236, SRR6171237, SRR6171238, SRR6171239, SRR6171240, SRR6171241, SRR6171242, SRR6171243, SRR6131113, SRR6131114, SRR6131115, SRR6131116, SRR6131117, SRR6131118 [70], SRR5710702, SRR5710701, SRR5710700, SRR5710699, SRR5710698, SRR5710697, ERR105067, ERR105069, ERR105073 and ERR105075, accession number at S6 Table) were extracted from the NCBI SRA database to determine the gene expression of the *PeD-J* genes and related JRL and DIR genes during different hormone treatments and development stages. The two hormone treatments included Gibberellin (GA) and naphthaleneacetic acid (NAA) (5 μM). The tissues included bamboo roots, rhizome, panicles and leaves, which were sampled when the plants’ germination was 20 cm, 50 cm and 100 cm tall, respectively. Paired reads was mapped to the Moso bamboo genome with TopHat2 [71]. Then, gene expression was computed by the Cufflinks program [72, 73]. Gene expression profiles were calculated by transcripts per million reads (TPM). Logarithm base 2 of each TPM value was utilized to draw the gene expression heat map using the Amazing Heatmap module in TBtools.

### Plant materials and stress treatment

Moso bamboo seeds were collected from Guilin, Guangxi Province, China. The seeds were pre-treated for 3 days at 4°C for vernalization before sowing them directly in sterilized potting soil. Seedlings were then cultivated in an experimental greenhouse at 22°C with a constant light cycle (16h light/8h dark) for 2 months. Roots, stems, young leaves and mature leaves were sampled. For methyl jasmonate (MeJA) hormone treatment: 100 μM MeJA solution was sprayed on the plant leaf surface [74]. Leaves were then sampled at 0 h, 3 h, 6 h, 12 h, 24 h, 48 h and 72 h after spraying. For abiotic stresses, the seedlings were kept at 4°C for cold treatment, watered with 20% PEG solution for drought treatment and treated with 200 mM NaCl solution for salt treatment. After 8 hours of treatment, the roots, stems, young leaves and mature leaves were sampled. All samples were immediately frozen in liquid nitrogen and then maintained at -80°C until RNA extraction and isolation.

### qRT-PCR analysis of genes

For quantitative RT-PCR (qRT-PCR), we used RNAprep Pure Plant Kit (Tiangen Company) to extract RNA from Moso bamboo samples, and used Prime-Script™ RT Kit (TaKaRa) to synthesize first strand cDNA from RNA according to the manufacturer’s manuals. Gene-specific qRT-PCR primers were designed to amplify each CDS by AllelID7 (http://sd.downxia.com/down/AlleleIsakdhad.rar). qRT-PCR was performed with SYBR Premix Ex Taq II reagent (TaKaRa) and was repeated at least three times on a CFX96 Real-Time System (BioRad). The bamboo *actin* gene was used as an internal control for normalization. PCR conditions were as follows: 95°C for 30s, followed by 39 amplification cycles of 95°C for 5s, and 60°C for 30s. The expression level for each gene was determined via the 2^(-ΔΔCT)^ method. Statistical analyses were performed using GraphPad Prism 8 software (GraphPad Software, Inc., La Jolla, CA), and one-way ANOVA was performed on the data, followed by a Tukey test. Where appropriate, data from individual samples were compared pairwise using the nonparametric Student’s t-test. Expression differences were considered significant with *P* values of less than 0.05.

### Sub-cellular localization analysis of *PeD-J03*

Total RNA from Moso bamboo samples was used for cDNA synthesis. The full-length cDNA of the *PeD-J03* gene (without the stop codon) was isolated by reverse transcription PCR (RT-PCR) using sequence-specific primers (S4 Table) and cloned into the pCambia1300 vector using a seamless cloning method. In this vector, the *PeD-J03* gene was fused to the green fluorescent protein (GFP) gene under the control of the cauliflower mosaic virus (CaMV) 35S promoter. The resulting 35S::PeD-J03-GFP plasmid was used for subcellular localization analysis and Agrobacterium-mediated transformation. The 35S::GFP of pCambia1300 empty vector was used as a control.

Agrobacterium tumefaciens (GV3101) containing the fusion construct was obtained by continuous incubation of rifampicin and kanamycin (0.05 g/ml) in Luria-Bertani (LB) medium at 28° C for 24h-48h. The bacterial cells were centrifuged and resuscitated in an invasive solution (10 mm MES, 0.1 mm Acetosyringone, 10 mm MgCl2) to a final OD600 = 0.5. A disposable needleless syringe was used to inject into the underside of tobacco leaves. The plants infiltrated with Agrobacterium tumefaciens were grown for 24 h at 23°C in the dark and then for an additional 24–72 h under a 16h light:8h dark cycle, after which the leaves were separated and the GFP signal was observed under a confocal microscope (Germany, Zeiss, LSM880).

## Results

### Identification and characterization of *PeD-J* genes in Moso bamboo

The Hidden Markov Model (HMM) profile of the jacalin domain (accession no. PF01419.17) was retrieved from the Moso bamboo genome to identify jacalin gene family members in Moso bamboo, and 25 family members were identified which had E-values less than or equal to 10^−10^. Next, the DIR domain (PF03018.11) model was searched and 38 DIR family members were identified with E values less than or equal to 10^−10^. Venn diagram analysis was used to identify four genes that were contained in both lists, which were considered *PeD-J* genes (S1 Fig). Based on their location on the chromosomes of Moso bamboo, we renamed these genes *PeD-J01*-*PeD-J04*. The sequence characteristics, localization and physicochemical properties of the D-J genes were then predicted (Table 1). Of the four PeD-J proteins, PeD-J04 was the smallest, with 304 amino acids (aa), and *PeD-J02* was the largest (345 aa). The molecular weight (MV) of the proteins ranged from 32.41 KDa to 37.62 KDa, and the isoelectric points (PIs) were between 5.90 and 9.39. The predicted grand average of hydropathicity (GRAVY < 0) for the majority of the D-J members revealed hydrophilicity. Each protein was predicted to have an alpha helix, beta turn, random coil and extended strand regions. All PeD-J proteins were predicted to lack a signal peptide and therefore were likely located in the cytoplasmic or nuclear compartment. Despite the lack of a predicted signal peptide, ApoplastP did predict that two proteins could be apoplastic.

**Table 1:** Physiochemical properties and secondary structural properties of *PeD-Js* genes.

Gene id	Gene name	Chromosome location	Gene start	Gene end	Strand	Molecular weight(kD)	Theoretical pI	Number of amino acids(aa)	Aliphatic index	Subcellular localization	Signal peptide	Alpha helix(%)	Beta turn(%)	Extended strand(%)	Random coil(%)
PH02Gene23777.t1	*PeD-J01*	scaffold7	63598179	63600076	+	33.71	9.22	308	82.56	Non-apoplastic	no	0.1169	0.0747	0.3182	0.4903
PH02Gene23778.t1	*PeD-J02*	scaffold7	63606614	63608798	+	37.62	9.39	345	76.23	Non-apoplastic	no	0.1478	0.0754	0.2928	0.4841
PH02Gene03445.t1	*PeD-J03*	scaffold12	2328758	2330820	+	32.97	5.90	306	78.63	Apoplastic	no	0.1078	0.0556	0.3301	0.5065
PH02Gene07832.t1	*PeD-J04*	scaffold22	36027877	36029694	-	32.41	7.00	305	82.39	Apoplastic	no	0.1082	0.0721	0.3311	0.4885

### Chromosome location and collinearity analysis of *PeD-Js*

**Fig 1:**
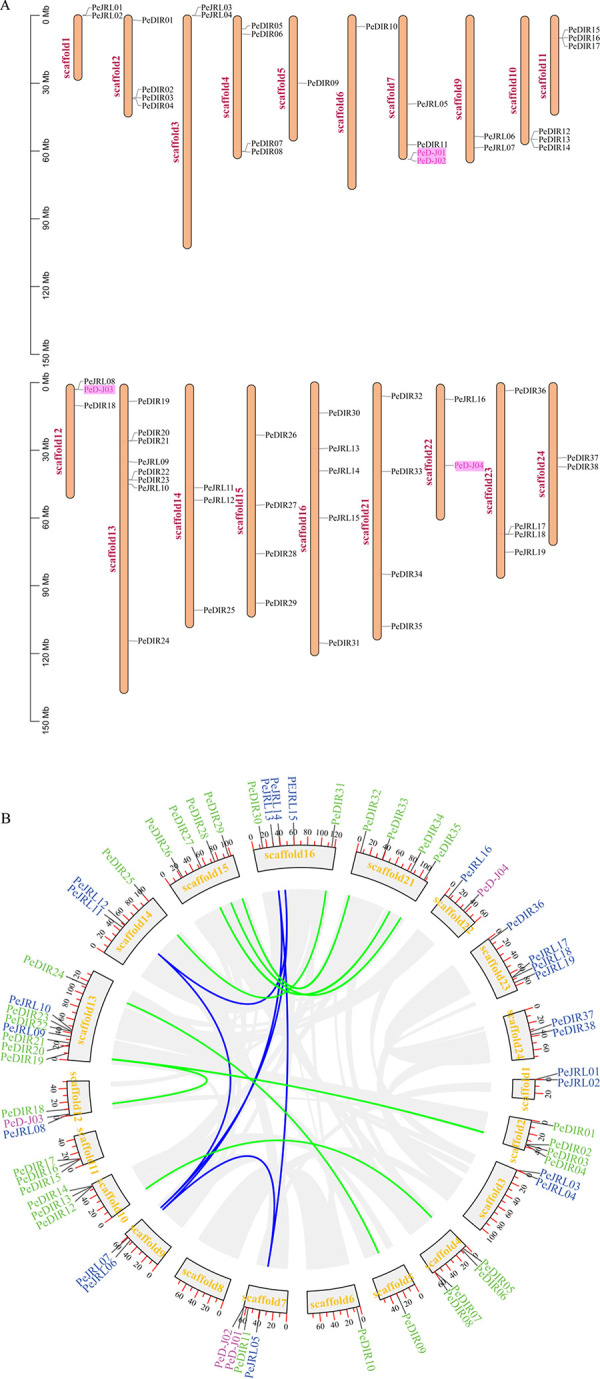
Chromosome location and collinearity analysis of *PeD-J* genes. A, Genomic distribution of the *PeD-J*, *PeDIR* and *PeJRL* genes on bamboo chromosomes. The *PeD-J* genes are numbered 1–4, *PeDIR* genes are numbered 1–38 and *PeJRL* genes are numbered 1–19. The scale represents the number of DNA bases. The chromosome number is shown on the left side of each strip, the gene name is on the right side of the chromosome and the purple rectangles represent *PeD-J* gene family members. B, Gene duplication among *PeD-Js*, *PeDIRs* and *PeJRLs* of bamboo. Green gene names represent the DIR gene family, blue gene names represent the JRL gene family and violet gene names represent the D-J gene family.

Next, *PeD-Js*, *PeJRLs* and *PeDIRs* were mapped to the corresponding chromosome scaffolds according to the GFF3 file (Fig 1A). Location analysis revealed that these genes were non-randomly located on chromosomes, with *PeD-Js* located on scaffold 7, 12 and 22. Scaffold 7 contained the largest number of *PeD-J* genes, and there was only one gene located on scaffold 12 and scaffold 22. *PeD-J03* was found to be located on scaffold 12 adjacent to the *PeJRL08* gene. There was no apparent correlation between the chromosome scaffold length and *PeD-J* gene distribution. Moreover, only *PeD-J01* and *PeD-J02* were found to form gene clusters on scaffold 7. To investigate the homologous loci relationships of DIR genes, JRL genes and PeD-J genes, collinearity analysis was performed (Fig 1B). This analysis showed that some DIR and JRL genes exhibited tandem and segmental duplications, while *PeD-J* genes did not have any duplication. This suggests that the *PeD-J* genes did not result from DIR or JRL gene family duplication.

### Gene family classification and phylogenetic tree construction of Moso bamboo

**Fig 2:**
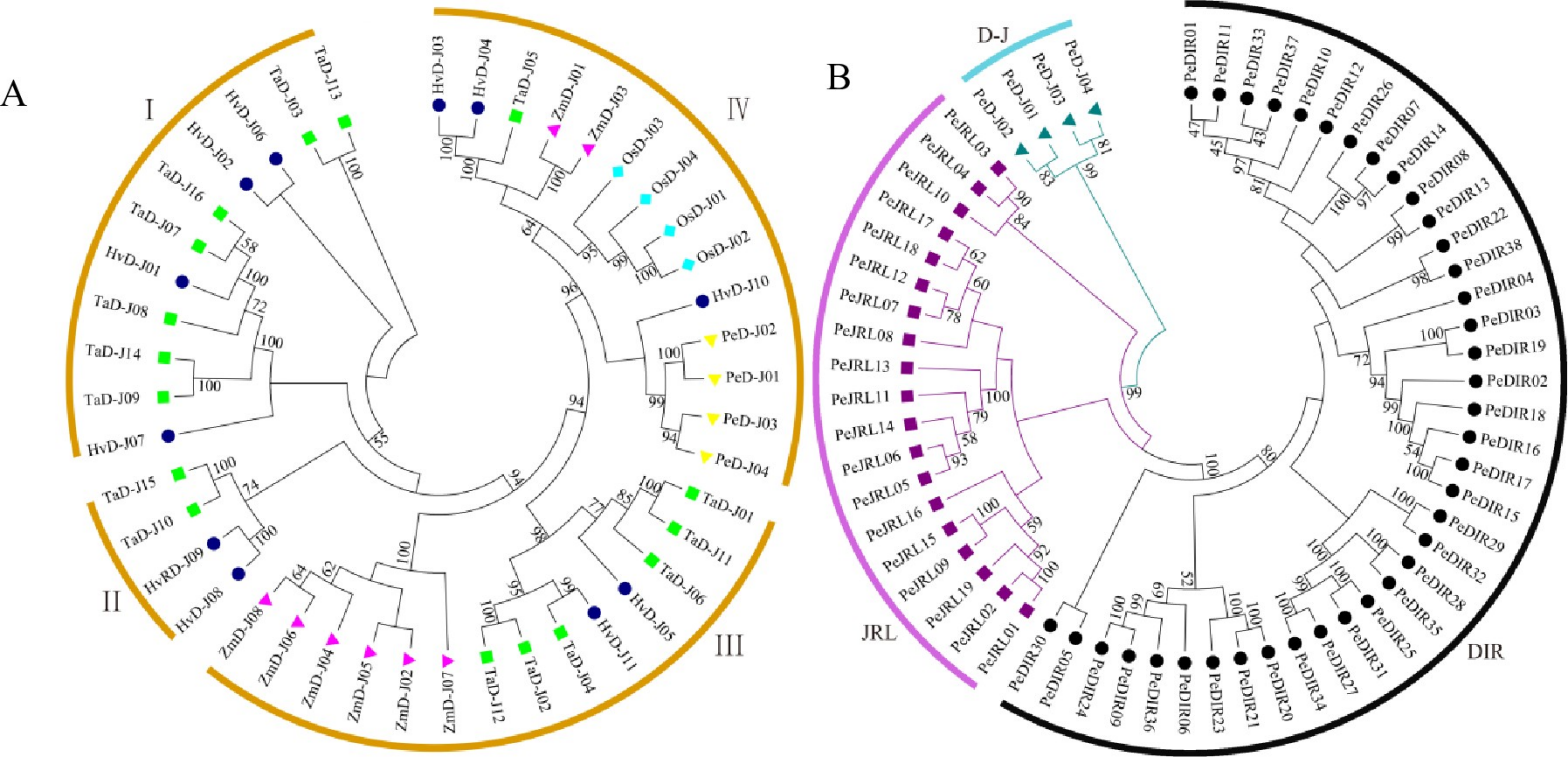
The phylogenetic tree of intraspecific and interspecies relationships. Phylogenetic trees without roots were constructed using the NJ method in MEGA 7.0. Bootstrap values from 1,000 replicates were indicated at each node. A, The phylogenetic relationships of bamboo (yellow squares), rice (lightblue diamonds), wheat (green squares), barley (darkblue circles) and maize (purple triangles) D-J sequences. Clustering resulted in a tree that is divided into four subfamilies: HvD-Js represent the D-J sequences of barley, OsD-Js represent the D-J sequences of rice, PeD-Js represent the D-J sequences of bamboo and TaD-Js represent the D-J sequences of wheat. B, The inter-species phylogenetic tree of bamboo is divided into three subfamilies, including DIR (black circles), JRL (darkpurple squares) and D-J (blue triangles).

To determine the phylogenetic relationship of D-J proteins from bamboo and other plants (rice, wheat, barley and maize), phylogenetic trees were constructed with the neighbor-joining (NJ) method. The D-J proteins were grouped into four distinct clades, named I to IV (Fig 2A). Most of the D-J proteins from wheat and barley were clustered together, while rice and bamboo each formed their own branches. Homology modeling showed that the four Moso bamboo genes were related to one barley gene. The phylogenetic tree of PeD-J proteins was constructed by MEGA 7.0. All protein sequences of the gene families were grouped into three subfamilies, with the chimeric D-J clustering into one group, and all classical JRLs and DIRs clustering into another two sets. A phylogenetic analysis (Fig 2B) indicated that the D-J and JRL gene families were closely related to the PeD-J cluster, which was divided into one subbranch.

### The genetic structure, motifs, conserved domains and sequence alignments of *PeD-J* genes

**Fig 3:**
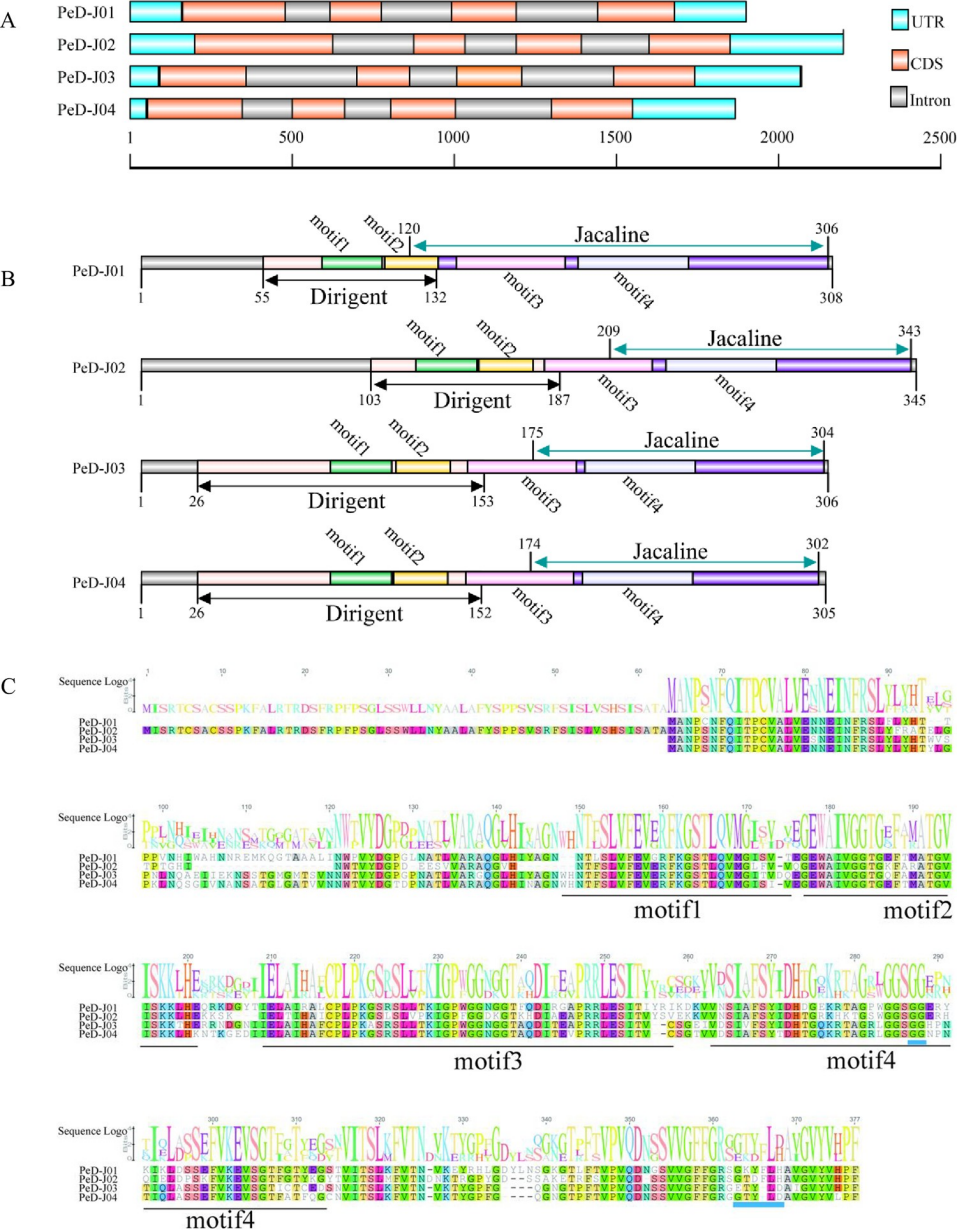
Genetic structures, conserved domains and multiple sequence alignment. A, Gene structure. The scale in Figure A represents the length of the nucleotide sequence. UTR, CDS and introns are represented by different colored regions. B, Conserved domains and motifs. The gray bar represents protein sequence length, the number represents the amino acid position. The dirigent domain is composed of the pink region, motif 1 and motif 2, and the jacalin structural domain is composed of the purple region, motif 3 and motif 4. the green region represents motif1, the yellow region represents motif2, the violet region represents motif3 and the lavender region represents motif4. C, Multiple sequence alignment of the PeD-J protein sequences. The number over the sequence indicates the position of the amino acids in the D-J proteins, in this figure. The main motifs that form each domain are outlined in horizontal lines, the motif site is shown below the alignment and the amino acid composition is shown above the alignment. The names of protein sequences are shown on the left side and the amino acids marked in blue represent the binding sites of sugars.

The evolutionary features and structural diversity of the D-J genes in bamboo were explored by examining their exon-intron structures. All *PeD-Js* possessed three introns as well as UTRs (Fig 3A). Additionally, D-J family proteins all contained two conserved domains and four motifs (Fig 3B). Motif1 and motif2 constituted the 130 amino acid N-terminal dirigent domain, while motif3 and motif4 constituted the 130 amino acid C-terminal jacalin domain. Next, four D-J genes were selected for sequence comparison. Their sequence similarity was found to be more than 65%, and all four proteins contained all four motifs. Notably, the PeD-J04 protein also had unique features, including GG and G-X3-D (G-X2-LD) carbohydrate motifs (Fig 3C).

### Homologous modeling of PeD-J protein structures

**Fig 4:**
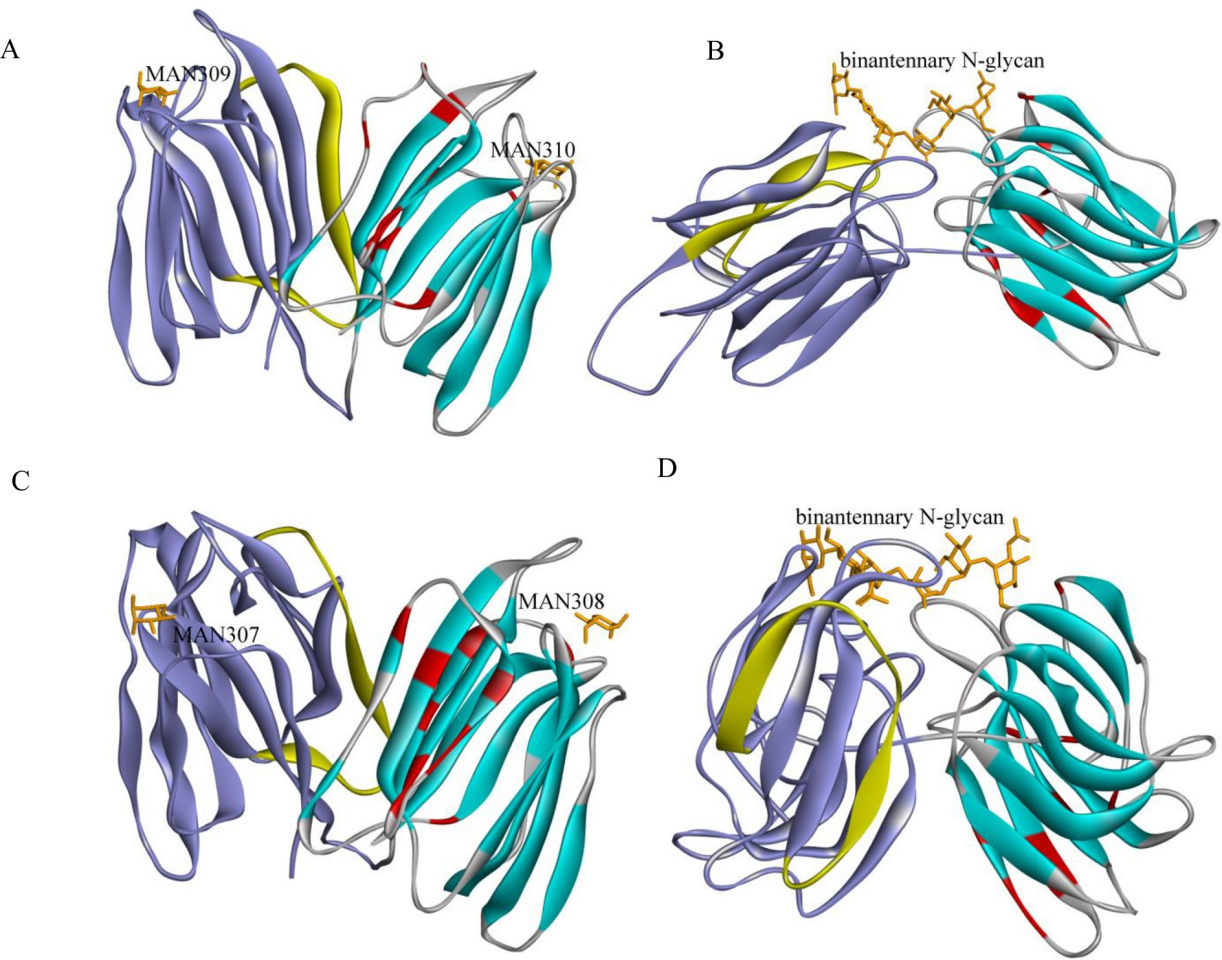
Protein structures based on homologous modeling. Protein structures are predicted based on sequence homology to known structures. The region of the substrate-binding is marked. The yellow chain represents the conserved amino acid residues of the dirigent domain region. The red chain represents the conserved amino acid residues involved in β-prismatic folding of the jacalin domain region and the orange chain represents the attached substrates in the substrate-binding center. A, PeD-J01 with jacalin-dirigent domain (blue and purple, PDB: 5GVY), which binds to MAN. B, PeD-J04 with a jacalin-dirigent domain (blue and purple, PDB: 5XFH), which binds to NAG, MAN, BMA or GAL. C, PeD-J03 with a jacalin-dirigent domain (blue and purple, PDB: 5GVY), which binds to MAN. D, PeD-J04 with a jacalin-dirigent domain (blue and purple, PDB: 5XFH), which binds to NAG, MAN, BMA or GAL.

After searching the PDB database, PeD-J01 and PeD-J03 proteins were found to have the highest similarity to 5gvy. PeD-J02 and PeD-J04 proteins were both found to have the highest degree of similarity to 5xfh. PeJRL12 of the JRL family and PeDIR08 of the DIR family were selected for homology modeling using the structural templates 1xxq, 6ood, 5xfh and 5gvy. As described in Fig 4, the tertiary structures of the *PeD-J* genes were predicted to be a heterozygous dimer with conserved jacalin (blue) and dirigent (purple) domains. The tertiary structure of the PeJRL12 and PeDIR08 proteins only had one domain (S2 and S3 Figs). Structural analysis revealed that proteins containing a DIR domain possessed a three-dimensional structure consisting of eight reverse-parallel chains in a β barrel-shaped structure, while all jacalin domain proteins have a β-prism fold structure formed by three-stranded β-sheets. For validation of the structural model, conserved amino acid residues (Glyl9, Gly22, Gly26, Gly93, Ser114, Gly 116, Gly120, Pro121, Gly123, Phel31, Gly142 and Phel43) in the JRL domain of PeJRL12 were compared to those found in PeD-J01, PeD-J02, PeD-J03 and PeD-J04. After comparison, these jacalin domain amino acids were found to be highly conserved (S3 Table).

The DIR domain of the PeD-J family proteins also contained the conserved motif V, which was comprised of PeD-Js. Similar conserved amino acids were found in other members of the PeD-J family (S3 Table). The type and distribution of the substrate-binding regions (orange) of the D-J family genes were significantly different from other families as well. In PeD-J02 and PeD-J04, these regions were predominantly distributed in hydrophobic regions at the junction of dirigent and jacalin domains. These regions were predicted to bind N-glycan, a polysaccharide condensate, which includes N-Acetyl-D-Glucosamine (NAG), Alpha-D-Mannose (MAN), Beta-D-Mannose (BMA) and Beta-D-Galactose (GAL). However, the substrates of PeD-J01 and PeD-J03 were mainly predicted to be Alpha-D-Mannose (MAN).

### Promoter analysis of *PeD-J* genes

**Fig 5:**
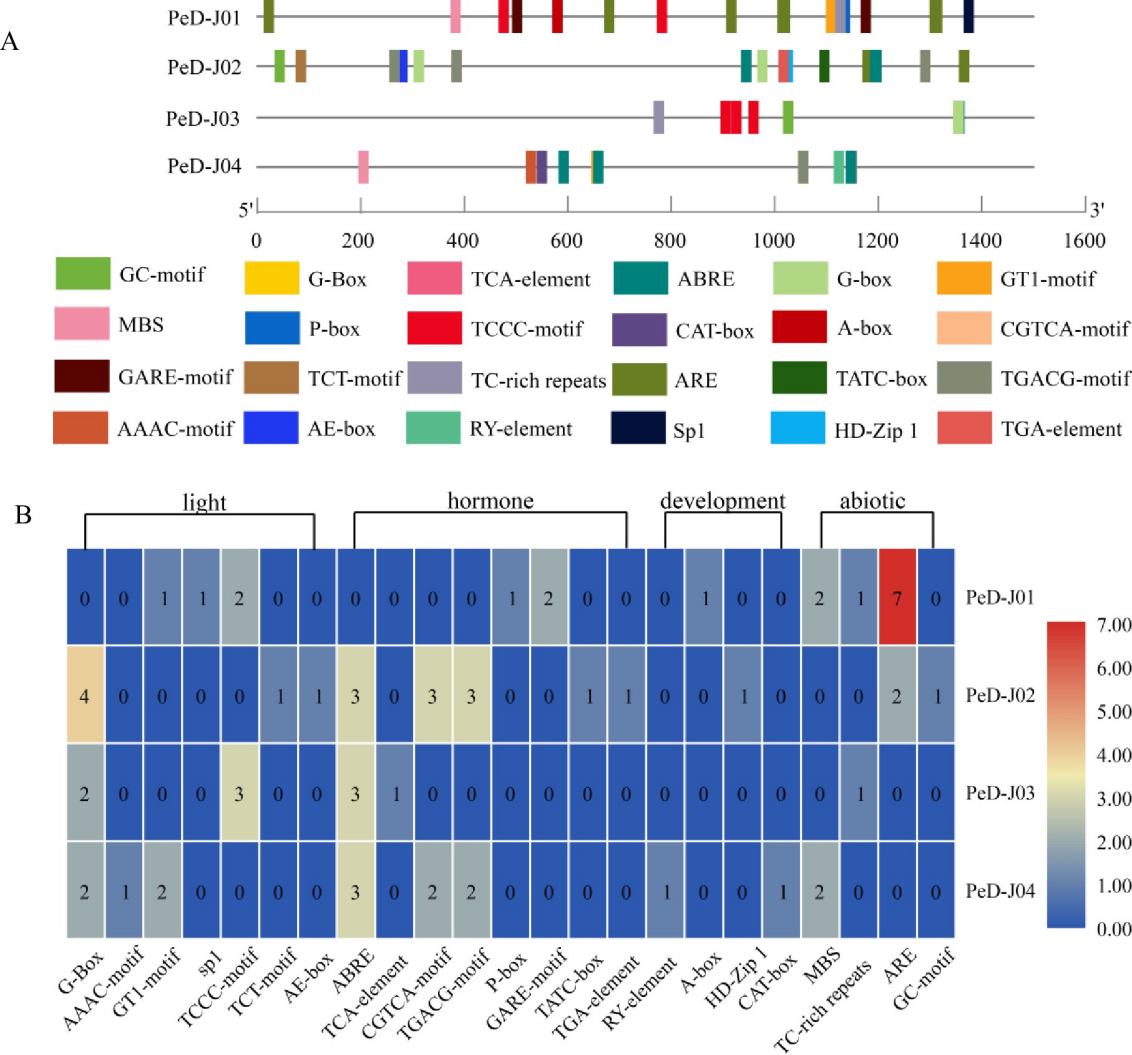
Promoter *cis*-element analysis of *PeD-J* genes. Promoter *cis*-element analysis of *PeD-Js*. The 1500 bp DNA fragments upstream of the ATG are analyzed using the online analysis software PlantCARE. Different *cis*-acting elements of D-J genes are displayed. A, Promoter position information. The different colored markers indicate different predicted *cis*-acting elements. B, Promoter number analysis. The color scale to the right of the heat map represents the number of promoters.

Promoters play a critical role in the initiation of gene transcription. To further understand the potential roles of *PeD-Js* in bamboo and how their expression is regulated, *cis*-acting elements of D-J gene promoters (1500 bp sequences upstream of ATG) were predicted with PlantCARE software. In total, 23 potential *cis*-acting elements involved in hormone-responsiveness (such as MeJA, abscisic acid, gibberellin, salicylic acid and auxin), abiotic-responsiveness, light-responsiveness and development regulation were identified in D-J promoters (Fig 5). Various light-responsive elements, such as a AAAC-motif, GT1-motif, TCCC-motif, G-Box, TCT-motif, Sp1-binding site and AE-box, were also found in D-J promoters. The *PeD-J02* promoter also harbored four G-Box elements. ABRE, the ABA responsiveness elements, could be found in the promoter regions of three *PeD-J* genes. There were three ABREs among genes with this element, which were found in *PeD-J02*, *PeD-J03* and *PeD-J04*, but not *PeD-J01*. Gibberellin-responsive (GA) elements included a P-box, GARE-motif and TATC-box. The CGTCA-motif and TGACG-motif are MeJA-responsive elements, which were predicted in the promoters of *PeD-J02* and *PeD-J04*. The TCA-element (only found in the *PeD-J03* promoter) is a salicylic acid (SA) responsive element.

The TGA-element, which has been shown to be involved in auxin responsiveness, was present in the *PeD-J02* promoter. TC-rich repeats have been associated with defense and stress responsiveness and were found in the promoters of *PeD-J01* and *PeD-J03*. MBS is a drought-inducibility element, which was found in the promoters of *PeD-J01* and *PeD-J04*. Regulatory elements essential for the anaerobic induction (ARE) were also found in two promoters, with *PeD-J01* containing seven ARE elements, and *PeD-J02* containing two ARE elements. Only *PeD-J02* was identified as having an element related to anoxic specific inducibility (GC-motif). The RY-element of *PeD-J04* was associated with seed-specific regulation, while the CAT-box of *PeD-J04* was related to meristem expression. HD-Zip 1 of the *PeD-J02* element is predicted to be related to differentiation of the palisade mesophyll cells and the A-box regulatory element was found in *PeD-J01*. Overall, these results indicated that the diverse *cis*-acting elements might participate in the regulation *PeD-J* gene family members, with impacts on developmental control, light response, hormone regulation and abiotic stresses.

### Expression patterns of the *PeD-J* genes

**Fig 6:**
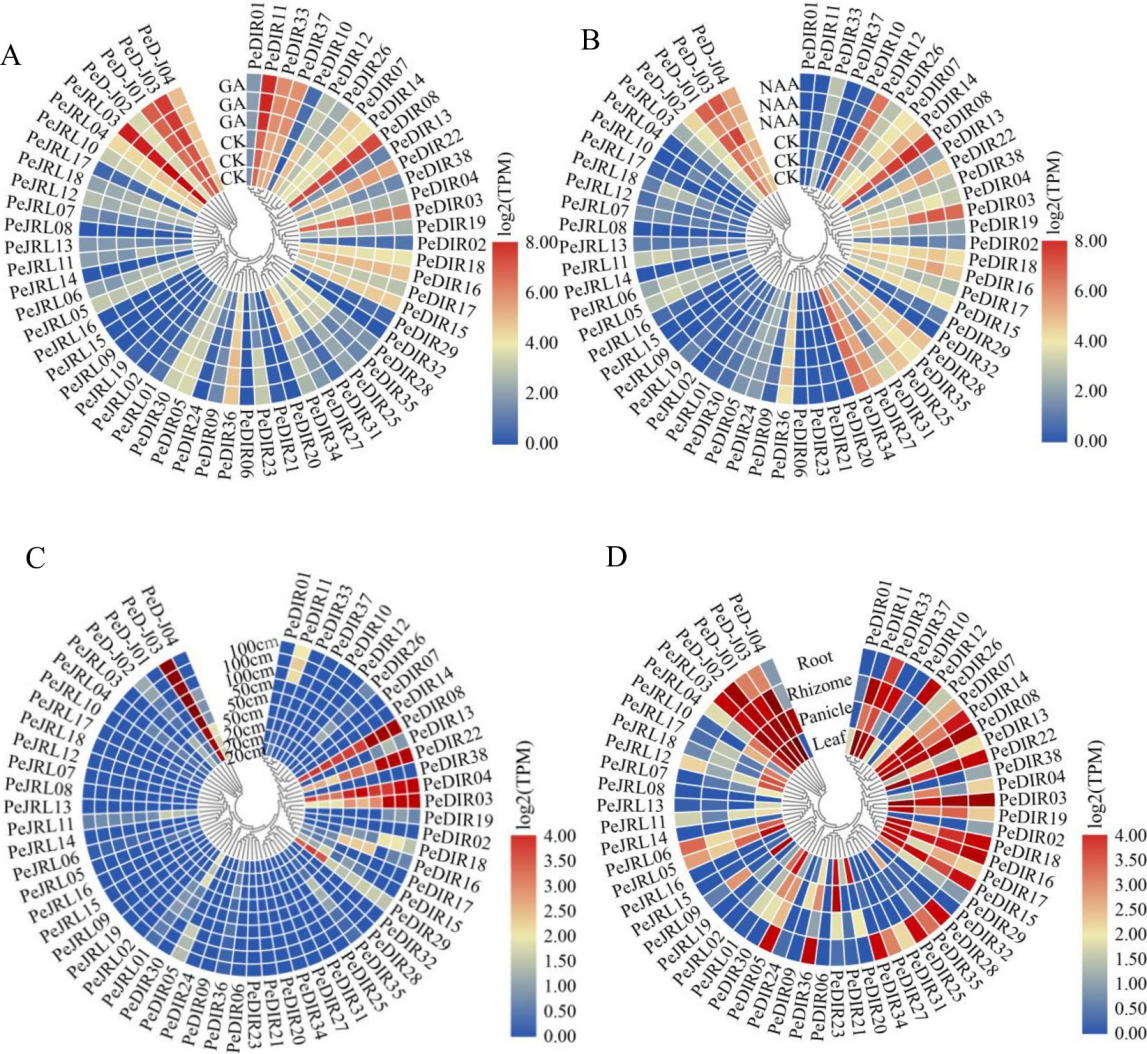
The expression profiles of *PeD-J* genes. A, Expression profile of *PeD-J* genes after GA treatment. B, Expression profile of *PeD-J* genes after NAA treatment. C, Expression profile of *PeD-J* genes in the shoots of bamboo at different growth heights. D, Expression profile of *PeD-J* genes in different tissues. CK represents the control group. Each group has three replicates. The relative expression levels are depicted according to the color scale, where a change from blue to red indicates transcript abundance from low to high. Gene expression is calculated as the sum of the abundance of all transcripts produced by a given gene.

The availability of a relevant transcriptome database makes it possible to study the gene expression of *PeD-J* genes in a comprehensive manner. To explore the potential functions of D-J genes in Moso bamboo, their expression was assessed in seedlings after treatment with different hormones (GA, NAA), at different growth heights (20, 50, 100 cm) and in different tissues (root, rhizome, panicles, leaf). Expression heat maps were drawn using TBtools. Under GA or NAA treatment (Fig 6A and 6B), there were no significant differences in the expression levels of *PeD-J02* and *PeD-J04*, while the expressions of *PeD-J01* and *PeD-J03* were up-regulated. Under GA treatment, there was an increase in the expression of *PeDIR07*, *PeDIR14* and *PeDIR23* and a decrease in the expression of *PeDIR12*, *PeDIR04*, *PeDIR28*, *PeDIR35*, *PeDIR25*, *PeDIR27* and *PeDIR34*. Under NAA treatment, the expression of *PeDIR14*, *PeDIR03*, *PeDIR22* and *PeDIR35* was up-regulated. In the D-J family, only *PeD-J03* was highly expressed at the bud development stage (Fig 6C). In the DIR family, five genes were highly expressed, four of which (*PeDIR08*, *PeDIR22*, *PeDIR04* and *PeDIR03*) were most strongly expressed at the top of the 100 cm stem. In the JRL family, all genes had low expression, which suggested that *PeD-J* and *PeDIR* genes may play a role in the high growth development of shoots. The expression of *PeD-J* family genes was also found to be tissue-specific. For example, the *PeD-J04* gene was highly expressed only in panicles, while *PeD-J02* was highly expressed in panicles, leaves, roots and rhizomes (Fig 6D). However, *PeD-J03* and *PeD-J01* were not highly expressed in roots but were found in other tissues.

**Fig 7:**
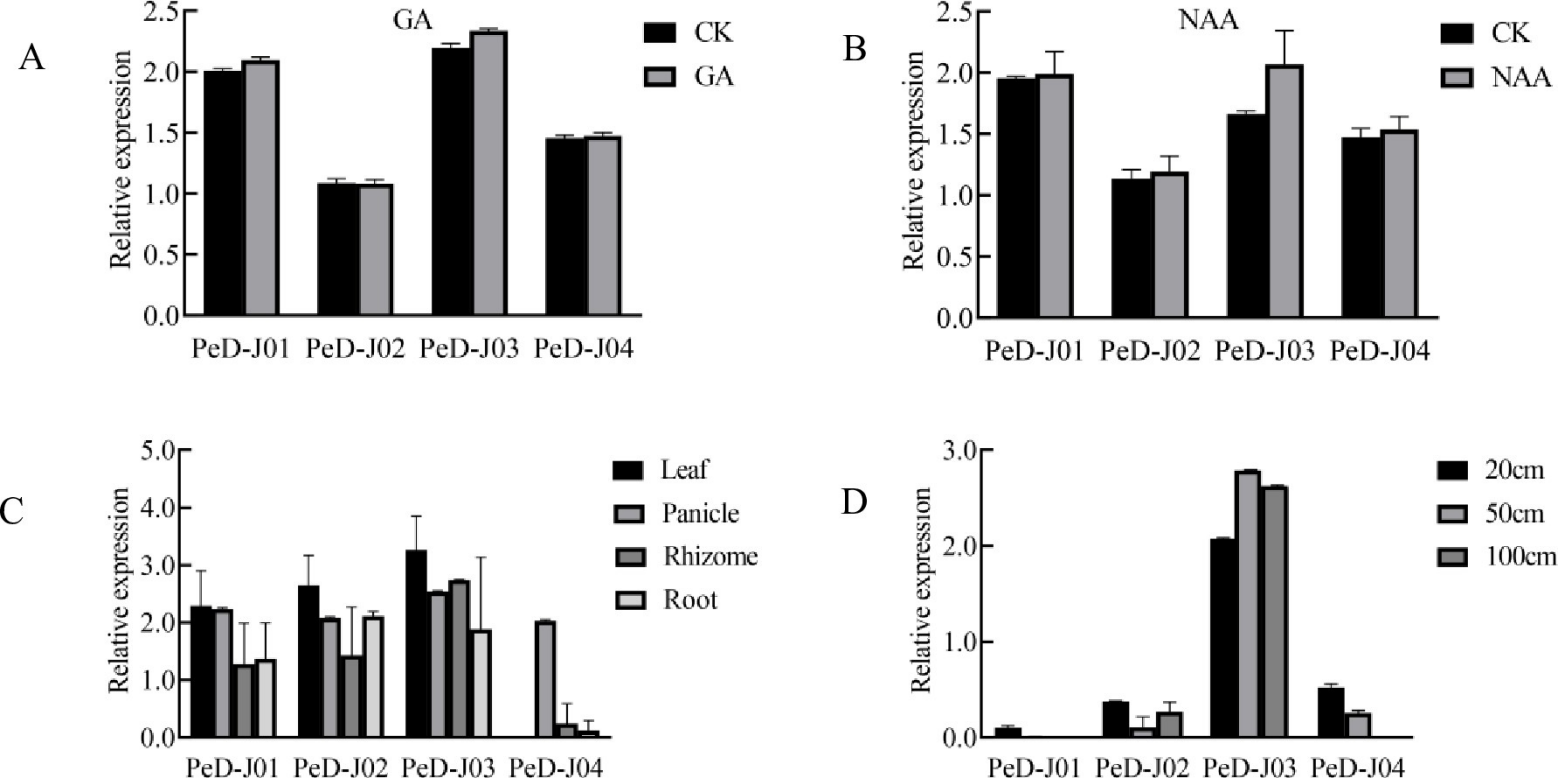
Expression patterns of *PeD-J* genes. The relative expression patterns of *PeD-J* genes are normalized as transcripts per million (TPM) and expressed as log values. A, Expression pattern after GA treatment. B, Expression pattern after NAA treatment. C, Expression pattern in different tissues. D, Expression pattern in shoots at different growth heights.

The expression patterns of Moso bamboo D-J genes during stress hormone response were investigated by construction of histograms. As could be seen from the figures, the gene expression level of the *PeD-J* genes was increased in seedlings treated with GA or NAA (Fig 7A and 7B). *PeD-J* family genes all had tissue-specific expression, indicating that they may play a role in the development of different tissues (Fig 7C). For example, *PeD-J04* was highly expressed in panicles but absent in leaves, while *PeD-J02/PeD-J03* were highly expressed in leaves and *PeD-J01/PeD-J03* were expressed at relatively low levels in the rhizomes. *PeD-J03*, on the other hand, was significantly expressed in rhizomes, panicles, roots and leaves.

Additionally, *PeD-J* genes showed differential expression over the course of shoot development (Fig 7D). *PeD-J03* was constitutively highly expressed, while the expression levels of *PeD-J01/PeD-J04* decreased during development, and *PeD-J02* fluctuated up and down over the course of development. At 20 cm of height, *PeD-J03* had the highest expression, followed by *PeD-J04* and *PeD-J02*, then *PeD-J01*. At 50 cm, *PeD-J03* had the highest expression, followed by *PeD-J04* and *PeD-J02*, while *PeD-J01* was not expressed at all. When bamboo reached 100 cm in height, *PeD-J01* and *PeD-J04* were not expressed, while gene *PeD-J03* had the highest expression, followed by *PeD-J02*. The above results indicated *PeD-J* genes were under strong developmental regulation.

### Differential expression of the *PeD-J* genes in different tissues and during abiotic stress and phytohormone treatments

**Fig 8:**
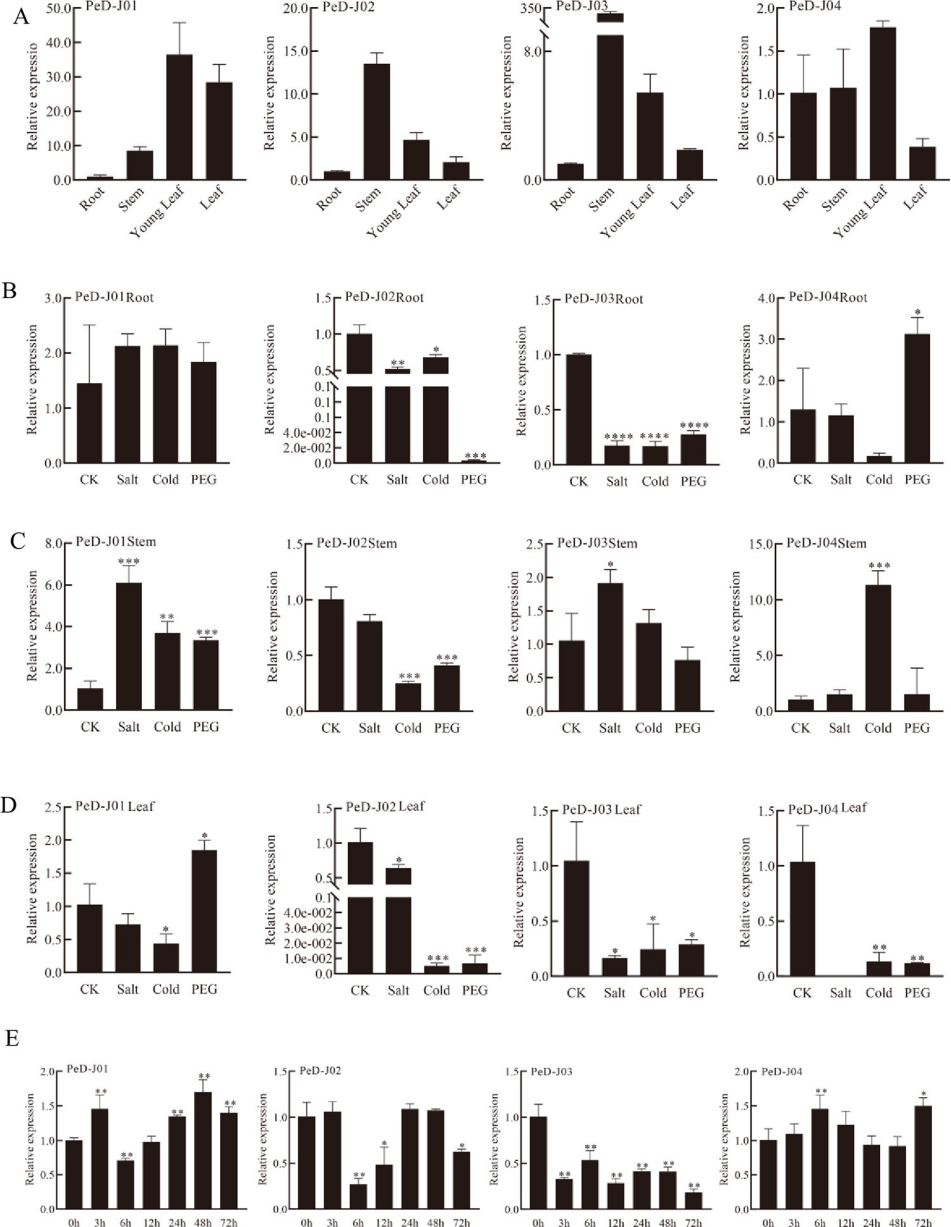
qRT-PCR analyses of *PeD-J* genes. A, Expression of *PeD-J*s in different tissues. B, Expression of *PeD-J*s in roots under abiotic stress treatment. C, Expression of *PeD-J*s in stems under abiotic stress treatment. D, Expression of *PeD-J*s in leaves under abiotic stress treatment. E, Expression of *PeD-J*s in leaves after MeJA treatment. 20% PEG solution was used to simulate drought. The concentration of salt and MeJA was 200 mM and 100 μM, respectively. Single asterisk indicates that the level of the gene expression in treatment group was significantly different from that in the control group (*t*-test, *p* < 0.05). Double asterisks indicate that there is a significant difference (*t*-test, *p* < 0.01). The relative expression levels of the target genes were examined using the 2^-ΔΔCt^ method. Error bars represent standard deviations of the means of three independent replicates.

The *cis*-elements predicted to be present in the promoters of *PeD-J* genes suggested that they are likely involved in growth, drought stress and hormone responses. To determine their expression patterns under different stresses and in different tissues, quantitative real-time PCR analysis was performed (S4 Table) to investigate the response of *PeD-Js’* expression to these treatments in two-month-old seedlings. This analysis revealed that *PeD-J01* and *PeD-J04* were highly expressed in young leaves, while *PeD-J03* was highly expressed in stems and *PeD-J02* was absent from nearly all tissues but expressed at a low level in mature leaves (Fig 8A). The expression levels of the D-J genes were analyzed in different tissues under high salinity, PEG, cold and normal conditions (Fig 8B– 8D). The abiotic stress results indicated that *PeD-J01* was up-regulated in roots under salt and cold stress, while the other three *PeD-J* genes were down-regulated. PEG stress resulted in increased expression of *PeD-J01* and *PeD-J04* in roots, while *PeD-J02* and *PeD-J03* were significantly down-regulated under the same conditions. In stems, the expression levels of *PeD-J01* and *PeD-J03* increased under salt stress, while the expression of *PeD-J04* was significantly up-regulated under cold stress. For the most part, stressful conditions resulted in reduced expression of *PeD-J* genes in leaves, with the exception of *PeD-J01*, which was induced under PEG stress. qRT-PCR was used to determine the expression levels of the four *PeD-Js* under MeJA treatments (Fig 8E). Two genes (*PeD-J01*/*PeD-J02*) were significantly down-regulated at 6h of MeJA treatment, while other genes were up-regulated. Interestingly, the expression levels of two genes (*PeD-J01*/*PeD-J03*) were significantly increased after MeJA treatment, and the expressions of most genes exhibited two peaks, with an initial rapid increase, followed by a later peak.

### Analysis of subcellular localization of the *PeD-J03* gene

**Fig 9:**
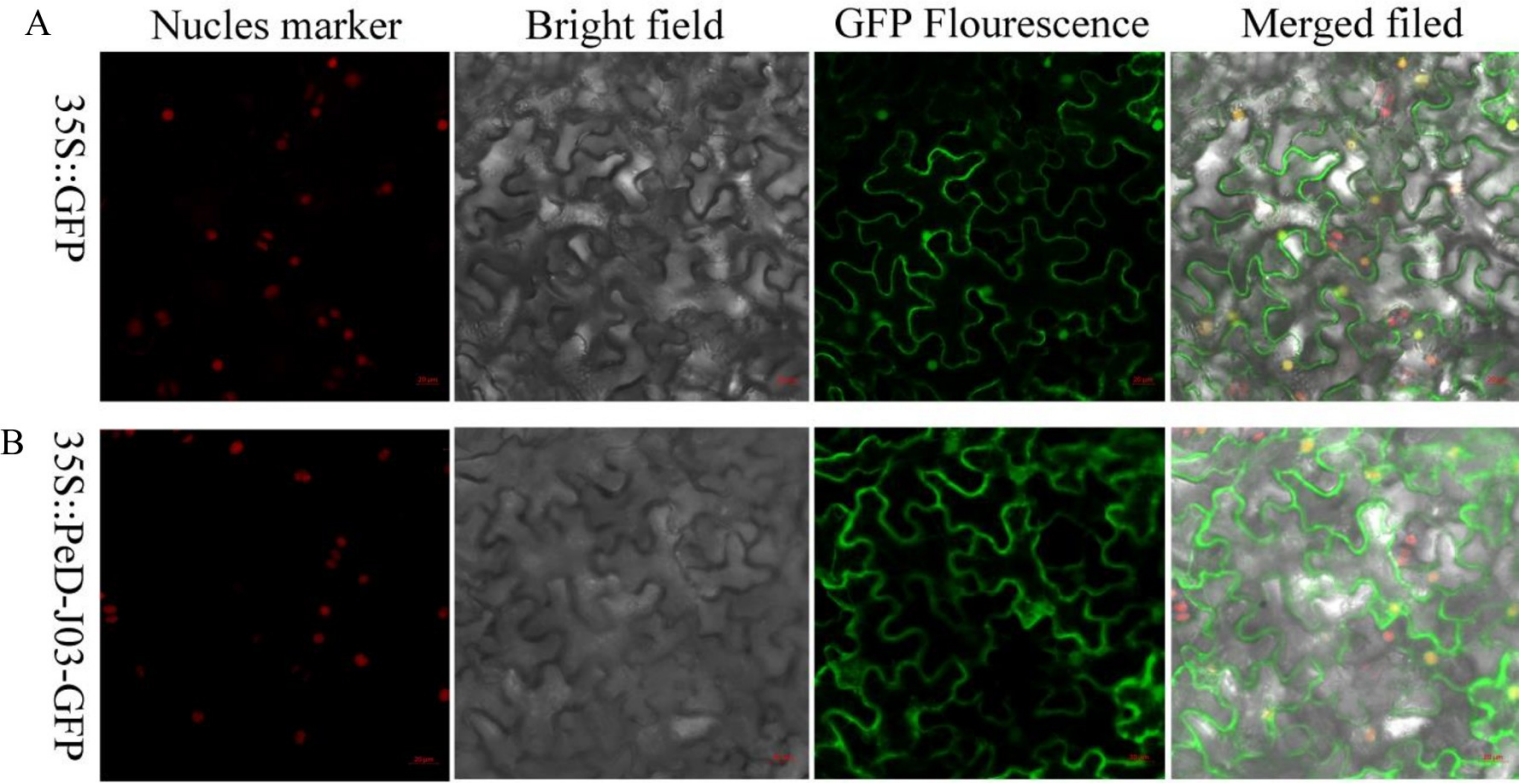
Subcellular localization analyses of 35S::GFP and 35S:: PeD-J03-GFP. A, Subcellular localization of 35S::GFP in tobacco leaves. B, Subcellular localization of 35S:: PeD-J03-GFP in tobacco leaves.

Transient expression of the 35S:: PeD-J03-GFP plasmid in tobacco epidermal cells was used to determine subcellular localization using an Agrobacterium infestation method. Compared to control 35S::GFP of pCambia1300 empty vector, 35S:: PeD-J03-GFP localized to cell membrane primarily, with a small amount in the nucleus (Fig 9).

## Discussion

Plant lectins are a class of proteins with glycan-binding properties and a wide range of functional diversity [75]. By interacting with various compound polysaccharides, lectins function as central mediators of information transfer in biological systems [76]. In the last few years, lectins have been shown to be mainly involved in binding to foreign glycans produced by plant pathogens, sometimes resulting in anti-nutritional or toxic effects on higher animals and insects [38]. Notably, most plant lectins specifically recognize exogenous polysaccharide compounds and are involved in endogenous regulation [37, 42, 77].

Dirigent-jacalin genes have been shown to play important roles in plant responses to drought stress in sugarcane. At least four DJ groups were identified in the sugarcane genome, which consists of seven non-redundant sequences [45], but the identity and expression patterns of *PeD-J* genes have rarely been studied in Moso bamboo at the genome-wide level. In this study, we identified four *PeD-J* genes in the genome of bamboo. We then performed a comprehensive analysis of the *PeD-J* gene family, including analysis of phylogeny and gene structures, conserved motifs, chromosomal locations and 3D structure, *cis*-element analysis and expression profiling. According to the inter-species phylogenetic tree, the D-J family of Moso bamboo was closely related to barley and the D-J family belonged to the same branch as the JRL family in the intra-species phylogenetic model. Studies have shown that a common ancestor of JRLs evolved before the divergence between monocotyledons and dicotyledons. The modern JRLs were formed by the duplication and tandem insertion of ancestral domains, and the formation of monocotyledon chimeric lectins occurred when a dirigent domain was inserted into cytoplasmic lectins [24, 46]. Closer examination revealed that chimeric jacarins are present in only a small fraction of monocotyledonous taxa and emerged from 50 to 25 million years ago [74]. The intraspecific evolutionary tree showed that JRLs were closely related to D-J genes, indicating that the two families may come from the same ancestor, which was consistent with previous studies.

All four *PeD-Js* have three exons and two introns, with an extremely similar overall structure. Additionally, the configurations of motifs recognized by MEME also reflected the conserved nature of *PeD-Js*, with similar motif structures and numbers. This high degree of conservation indicates a strong selection pressure throughout *PeD-J* gene family evolution.

The tertiary structure prediction results showed that the PeD-J03 protein and PeD-J04 protein have slightly different structures, but both possess a chimeric conserved hydrophobic cavity consisting of a barrel-shaped domain and β-prismatic folded structure. When Pickel et al. [50] determined the structure of the AtDIR6 protein of *Arabidopsis thaliana*, they found it was composed of eight reverse parallel chains in series, with a hydrophobic cavity in the middle. These findings indicated that this hydrophobic binding protein might have evolved from a DIR protein, while the tertiary structure of PeDIR08 predicted in this study was similar to that of AtDIR6. The JRL protein structure consisted of a β-prism structure composed of β-sheets [78], and the D-J protein was a chimeric tertiary structure composed of a DIR structure and a JRL structure. Bourne et al. [78] found a JRL protein structure with twelve residues (Glyl1 Gly14, Gly18, Gly84, Tyr102, Asn103, Gly108, Prol09, Gly111, Phel18, Gly129 and Phel30) that played a key role in the folding of the β-prismatic region in *Helianthus tuberosus*. Through 3D homologous modeling, these conserved amino acid residues (Glyl9, Gly22, Gly26, Gly93, Ser114, Gly 116, Gly120, Pro121, Gly123, Phel31, Gly142 and Phel43) in the JRL domain of the PeJRL12 protein were predicted to also be involved in β-prismatic folding. These residues were also found in PeD-Js, and the amino acid residues of the jacalin domain of PeD-Js were highly conserved. Ralph et al. [79] found five conserved motifs in DIR genes of spruce (motif I-V). Their modeling results indicated that the conserved amino acid residues of motif V were found in the dirigent protein of PeDIR08 (Val73, Val74, Gly75, Gly76, Thr77, Gly78, Asp79, Phe80, Phe81, Met82, Ala83, Arg84, Gly85, Val86, Ala87, Thr88 and Leu89) and also found in chimeric protein sequences of PeD-Js. It has been speculated that the mutation of a few conserved bases may be the cause of their substrate differences.

During multi-sequence comparison and modeling of PeD-J family proteins, conserved carbohydrate-binding sites were identified in jacalin region (S4–S7 Figs). In the G-X3-D (G-X2-LD) motif, one of the glycan-binding-related sites, the L residue in the X3 region, appears to be the most conserved in the taxon [80]. It was found that the sugar-binding sites of the PeD-J04 protein included a GG and a G-X2-LD motif, which was consistent with the second sugar-binding site found in BanLec of a JRL protein by Meagher et al. [80]. Additionally, the G-X2-LD structure of PeD-J04 binds the NAG in the biantennary N-glycan (S6 and S7 Figs). In general, hydrogen bond equilibrium and hydrophobic interactions are the dominant modes of lectin-carbohydrate interaction [81]. However, the loop structures can also affect the specificity of glycosyl binding [82]. Additionally, the conserved aspartic acid of the BL3 linking loop resides in the G-X3-D motif that controls the activity of the lectin and determines the lectin and carbohydrate recognition characteristics of the protein. In addition, a number of other conserved residues and specific amino acid residues have been identified, some of which are specifically involved in sugar binding and quaternary structure formation [83–85]. The other three genes with only the GG motif may have undergone base mutations during evolution, so that the sugar-binding site was abolished. Monocotyledonous chimeric lectins show a degree of diversity in terms of carbohydrate binding specificity. Most of the monocot chimeric lectin proteins studied (TaJA1, Ma et al., [51]; OsJVC1, Jiang et al., [42]; TaHfr1, Subramanyam et al., [44]) specifically bind mannose. Homology modeling indicated that PeD-J01 and PeD-J03 bind mannose, and may belong to the mannose-specific jacalin lectins (mJRLs). The same modeling indicated that PeD-J02 and PeD-J04 could bind either mannose or galactose of biantennary N-glycan (S6 and S7 Figs). The glycan-binding properties of these genes were similar to the classical jacalin genes, which was presumed to be more closely related to the JRL family, and the dirigent domain may have been inserted during later evolution [46].

PeD-Js with dirigent domains at the N-terminal and JRL domains at the C-terminal are characterized by a structure of highly conserved modular domains, and it has been shown that dirigent and jacalin domains are associated during evolution. The lectin domains in maize BGAF and sorghum SL exhibited significantly different glycan specificity compared with full-length proteins, since the loss of the N-terminal dirigent domains mainly affected glycan-binding specificity [41]. More recently, Ma et al. [51] found that transgenic plants containing *Ta-JA1*, which only has an N-terminal dirigent domain, could not exhibit agglutination activity and participate in disease resistance, but did have altered sensitivity to salt stress. However, *Ta-JA1* contained a unique dual domain, dirigent domain and jacalin domain, which could effectively agglutinate rabbit erythrocytes, leading to enhanced disease resistance against bacterial, fungal and viral pathogens. Van Damme [38] and Subramanyam et al. [25] found that wheat plants were responsive to infection of first-instar *Hessian fly larvae* by increasing the expression of the *Hfr-1* gene, which encodes a protein with a C-terminal jacalin domain and an N-terminal disease responsive dirigent domain. These results implied that protein fusions were functionally significantly enhanced and positively selected.

JRL fusions to other proteins may have been required for the development of new functions and previous research has found D-J genes which appear to be involved in responses to specific biotic or abiotic stimuli [25, 53, 76, 86]. These genes may therefore have evolved via extension of the jacalin domain in JRLs and the fusion of other functional domains that helped plants adapt to various environmental challenges [85].

A comprehensive expression profile of all *PeD-J* genes in bamboo suggested that *PeD-J03* played a role in the development of shoots and *PeD-J03* and *PeD-J04* responded to hormone stress. Furthermore, comprehensive analysis of gene expression profiles in different tissues identified numerous tissue-specific expression patterns. Chimeric lectins play an important role in plant responses to biotic and abiotic stresses [46]. In particular, *PeD-J* genes from D-J lectins were significantly induced under PEG, cold and salt stress. It has previously been shown that treatment with MeJA could induce lectin activity in the leaves of rice, barley, wheat and maize [76]. Studies have also shown that plant growth regulators, including phytohormones, could regulate plant responses to environmental stimuli [87, 88]. MeJA activates a series of signal transduction pathways involved in plant responses to abiotic and biotic stresses, thereby enhancing stress tolerance [89]. In this study, it was found that the four *PeD-J* genes responded differently to MeJA treatment [30, 90]. The expression profiles of *PeD-Js* in different tissues were analyzed by qRT-PCR, and *PeD-J02* was found to only be expressed in leaves, while the other three genes were expressed in roots, stems and leaves. Taken together, our results provide useful information for further research into the role that *PeD-J* genes play in development and stress response.

## Supporting information

S1 Table The DIR family renamed of Moso bamboo. (DOCX)

S2 Table The JRL family genes renamed of Moso bamboo. (DOCX)

S3 Table Conserved aa Sites of PeD-J family proteins. (DOCX)

S4 Table The primer of qRT-PCR of Moso bamboo and the primer of subcellular localization. (DOCX)

S5 Table The IDs and sequences of D-J proteins from rice, wheat, barley and maize using for phylogenetic tree. (DOCX)

S6 Table The accession numbers of transcriptome data. (DOCX)

S1 Fig The venn graph of PeDIR and PeJRL. The blue circle indicates DIR genes and the orange circle indicates JRL genes. The 4 *PeD-J* genes in the middle are crossovers.
(DOCX)

S2 Fig Protein structures based on homologous modeling of PeJRL12. The blue amino acids are conserved amino acid residues and the black amino acids are binding substrates.
(DOCX)

S3 Fig Protein structures based on homologous modeling of PeDIR08. The light purple amino acids are conserved amino acid residues in DIR domain.
(DOCX)

S4 Fig Protein structures based on homologous modeling of PeD-J02. The yellow chain represents the conserved amino acid residues of the dirigent domain region. The red chain represents the conserved amino acid residues involved in β-prismatic folding of the jacalin domain region.
(DOCX)

S5 Fig The 2D diagram of the binding site of PeD-J02. The diagram shows the important covalent bonds to which the substrate is bound as well as the important amino acid residues.
(DOCX)

S6 Fig Protein structures based on homologous modeling of PeD-J04. The yellow chain represents the conserved amino acid residues of the dirigent domain region. The red chain represents the conserved amino acid residues involved in β-prismatic folding of the jacalin domain region. The blue amino acid residues (GLY221, GLY222, GLY291, GLY292, THR293, TYR294, LEU295 and ASP296) are the second sugar binding site. NAG306 is the binding substrate.
(DOCX)

S7 Fig The 2D diagram of the binding site of PeD-J04. The diagram shows the important covalent bonds to which the substrate is bound as well as the important amino acid residues.
(DOCX)

## References

[1] Peng ZH, Zhang CL, Zhang Y, Hu T, Mu SH, Li XP, et al. Transcriptome Sequencing and Analysis of the Fast Growing Shoots of Moso Bamboo (*Phyllostachys edulis*). PLoS ONE. 2013b; 8(11):e78944. 10.1371/journal.pone.0078944 24244391 PMC3820679

[2] Li L, Cheng ZC, Ma YJ, Bai QS, Li XY, Cao ZH, et al. The association of hormone signalling genes, transcription and changes in shoot anatomy during moso bamboo growth. Plant Biotechnol J. 2018;16:72–85. 10.1111/pbi.12750 28499069 PMC5785349

[3] Pan F, Wang Y, Liu HL, Wu M, Chu WY, Chen DM, et al. Genome-wide identification and expression analysis of SBP-like transcription factor genes in Moso Bamboo (*Phyllostachys edulis*). BMC Genomics. 2017;18(1):486. 10.1186/s12864-017-3882-4 28655295 PMC5488377

[4] Ramakrishnan M, Yrjälä K, Vinod KK, Sharma A, Cho J, Satheesh V, et al. Genetics and genomics of moso bamboo (*Phyllostachys edulis*): Current status, future challenges, and biotechnological opportunities toward a sustainable bamboo industry. Food Energy Secur. 2020;9(4):e229. 10.1002/fes3.229

[5] Tao GY, Ramakrishnan M, Vinod KK, Yrjälä K, Satheesh V, Cho J, et al. Multi-omics analysis of cellular pathways involved in different rapid growth stages of moso bamboo. Tree Physiol. 2020;40: 1487–1508. 10.1093/treephys/tpaa090 32705116

[6] Peng Z, Lu Y, Li LB, Zhao Q, Feng Q, Gao ZM, et al. The draft genome of the fast-growing non-timber forest species moso bamboo (Phyllostachys heterocycla). Nat Genet. 2013a;45(suppl. 1):456–461. 10.1038/ng.2569 23435089

[7] Eggermont L, Stefanowicz K, Van Damme EJM. Nictaba homolecularogs from *Arabidopsis thaliana* are involved in plant stress responses. Front Plant Sci. 2017;8:2218. 10.3389/fpls.2017.02218 29375596 PMC5767604

[8] Hu ZJ, Lv XZ, Xia XJ, Zhou J, Shi K, Yu JQ, et al. Genome-Wide identification and expression analysis of Calcium-dependent protein Kinase in tomato. Front Plant Sci. 2016;7:469. 10.3389/fpls.2016.00469 27092168 PMC4824780

[9] Xu WW, Huang WC. Calcium-dependent protein Kinases in phytohormone signaling pathways. Int J Mol Sci. 2017;18(11):2436. 10.3390/ijms18112436 29156607 PMC5713403

[10] Zhou AM, Liu EH, Liu J, Feng S, Gong SF, Wang JG. Characterization of increased cuticular wax mutant and analysis of genes involved in wax biosynthesis in Dianthus spiculifolius. Hortic Res. 2018;5(1):296–302. 10.1038/s41438-018-0044-z 30083355 PMC6068182

[11] Wang ZM, Wang MY, Liu LK, Meng FJ. Physiological and proteomic responses of diploid and tetraploid black locust (*Robinia pseudoacacia L.*) subjected to salt stress. Int J Mol Sci. 2013;14: 20299–20325. 10.3390/ijms141020299 24129170 PMC3821616

[12] Wei H, Movahedi A, Xu C, Sun WB, Wang P, Li DW, et al. Characterization, expression profiling, and functional analysis of *PtDef*, a defensin-encoding gene from *Populus trichocarpa*. Front Microbiol. 2020;11:106. 10.3389/fmicb.2020.00106 32117134 PMC7018670

[13] Wang YJ, Jiang L, Chen JQ, Tao L, An YM, Cai HS, et al. Overexpression of the alfalfa *WRKY11* gene enhances salt tolerance in soybean. Plos ONE. 2018;13(2):e0192382. 10.1371/journal.pone.0192382 29466387 PMC5821330

[14] Jung IJ, Ahn JW, Jung S, Hwang JE, Hong MJ, Choi HI, et al. Overexpression of rice jacalin-related mannose-binding lectin (*OsJAC1*) enhances resistance to ionizing radiation in *Arabidopsis*. BMC Plant Biol. 2019;19(1):561. 10.1186/s12870-019-2056-8 31852472 PMC6921557

[15] Tan CM, Chen RJ, Zhang JH, Gao XL, Li LH, et al. *OsPOP5*, a prolyl oligopeptidase family gene from rice confers abiotic stress tolerance in *Escherichia coli*. Int J Mol Sci. 2013;14(10):20204–20219. 10.3390/ijms141020204 24152437 PMC3821611

[16] Van Holle S, Van Damme EJM. Distribution and evolution of the lectin family in soybean (*Glycine max*). Molecules. 2015;20(2):2868–2891. 10.3390/molecules20022868 25679048 PMC6272470

[17] Jiang SY, Ma Z, Ramachandran S. Evolutionary history and stress regulation of the lectin superfamily in higher plants. BMC Evol Biol. 2010;10(1):79. 10.1186/1471-2148-10-79 20236552 PMC2846932

[18] Balciunaite G, Haimi PJ, Mikniene Z, Savickas G, Ragazinskiene O, Juodziukyniene N, et al. Identification of *Echinacea Purpurea (L.)* moench root LysM lectin with nephrotoxic properties. Toxins (Basel). 2020;12(2):88. 10.3390/toxins12020088 32013058 PMC7076766

[19] Azarkan M, Feller G, Vandenameele J, Herman R, El Mahyaoui R, Sauvage E, et al. Biochemical and structural characterization of a mannose binding jacalin-related lectin with two-sugar binding sites from pineapple (*Ananas comosus*) stem. Sci Rep. 2018;8(1):11508. 10.1038/s41598-018-29439-x 30065388 PMC6068142

[20] Rauf I, Javaid S, Naqvi RZ, Mustafa T, Amin I, Mukhtar Z, et al. In-planta expression of insecticidal proteins provides protection against lepidopteran insects. Sci Rep. 2019;9(1):100–108. 10.1038/s41598-018-36614-7 31043622 PMC6494996

[21] Xiang Y, Song M, Wei Z, Tong J, Zhang L, Xiao L, et al. A jacalin-related lectin-like gene in wheat is a component of the plant defence system. Journal of Experimental Botany. 2011;62(15):5471–5483. 10.1093/jxb/err226 21862481 PMC3223046

[22] Peumans WJ, Van Damme EJM. Lectins as plant defense proteins. Plant Physiol. 1995;109(2):347–352. 10.1104/pp.109.2.347 7480335 PMC157596

[23] Van Holle S, Van Damme EJM. Signaling through plant lectins: modulation of plant immunity and beyond. Biochem Soc Trans Biochemical Society Transactions. 2018;46(2):217–233. 10.1042/BST20170371 29472368

[24] Van Damme EJM, Peumans WJ, Barre A, Rougé P. Plant Lectins: a composite of several distinct families of structurally and evolutionary related proteins with diverse biological roles. Critical Reviews in Plant Sciences. 1998;17(6): 575–692. 10.1080/07352689891304276

[25] Subramanyam S, Sardesai N, Puthoff DP, Meyer JM, Nemacheck JA, Gonzalo M, et al. Expression of two wheat defense-response genes, *Hfr-1* and *Wci-1*, under biotic and abiotic stresses. Plant Sci. 2006;170(1):90–103. 10.1016/j.plantsci.2005.08.006

[26] Jia XY, Xu CY, Jing RL, Li RZ, Mao XG, Wang JP, et al. Molecular cloning and characterization of wheat calreticulin (*CRT*) gene involved in drought-stressed responses. J Exp Bot. 2008;59(4):739–751. 10.1093/jxb/erm369 18349049

[27] Zhang WL, Peumans WJ, Barre A, Astoul CH, Rovira P, Rougé P, et al. Isolation and characterization of a jacalin-related mannose-binding lectin from salt-stressed rice (*Oryza sativa*) plants. Planta. 2000;210(6):970–978. 10.1007/s004250050705 10872230

[28] Abebe T, Skadsen RW, Kaeppler HF. A proximal upstream sequence controls tissue-specific expression of Lem2, a salicylate-inducible barley lectin-like gene. Planta. 2005;221(2):170–183. 10.1007/s00425-004-1429-9 15605240

[29] Jia F, Rock CD. Jacalin lectin *At5g28520* is regulated by ABA and miR846. Plant Signaling & Behavior. 2013;8(6):e24563. 10.4161/psb.24563 23603955 PMC3909087

[30] Vandenborre G, Groten K, Smagghe G, Lannoo N, Baldwin IT, Van Damme EJM. Nicotiana tabacum agglutinin is active against *Lepidopteran* pest insects. Journal of Experimental Botany. 2009;61(4):1003–1014. 10.1093/jxb/erp365 20018900

[31] Sankaranarayanan R, Sekar K, Banerjee R, Sharma V, Surolia A, Vijayan M. A novel mode of carbohydrate recognition in jacalin, a *Moraceae* plant lectin with a beta-prism fold. Nat Struct Biol. 1996;3(7):596–603. 10.1038/nsb0796-596 8673603

[32] Lee X, Thompson A, Zhang ZM, Ton-that H, Biesterfeldt J, Ogata C, et al. Structure of the complex of Maclura pomifera agglutinin and the T-antigen disaccharide, Galβ1, 3GalNAc. Journal of Biological Chemistry. 1998;273(14):6312–6318. 10.1074/jbc.273.11.6312 9497359

[33] Rougé P, Peumans WJ, Barre A, Van Damme EJM. A structural basis for the difference in specificity between the two jacalin-related lectins from mulberry (*Morus nigra*) bark. Biochemical and Biophysical Research Communications. 2003;304(1):91–97. 10.1016/s0006-291x(03)00538-2 12705889

[34] Van Damme EJM, Hause B, Hu J, Barre A, Rouge P, Proost P, et al. Two distinct jacalin-related lectins with a different specificity and subcellular location are major vegetative storage proteins in the bark of the black mulberry tree. Plant Physiol. 2002;130(2):757–769. 10.1104/pp.005892 12376642 PMC166604

[35] Nagano AJ, Fukao Y, Fujiwara M, Nishimura M, Hara-Nishimura I. Antagonistic jacalin-related lectins regulate the size of ER body-type beta-glucosidase complexes in *Arabidopsis thaliana*. Plant Cell Physiol. 2008;49(6):969–980. 10.1093/pcp/pcn075 18467340

[36] Peumans WJ, Hause B, Van Damme EJM. The galactose-binding and mannose-binding jacalin-related lectins are located in different sub-cellular compartments. FEBS letters. 2000;477(3):186–192. 10.1016/s0014-5793(00)01801-9 10908718

[37] Xing LJ, Li J, Xu YY, Xu ZH, Chong K. Phosphorylation modification of wheat lectin VER2 is associated with vernalization-induced O-GlcNAc signaling and intracellular motility. PLoS ONE. 2009;4:e4854. 10.1371/journal.pone.0004854 19287503 PMC2654674

[38] Van Damme EJM, Lannoo N, Peumans WJ. Plant Lectins. Adv Botanical Res. 2008;48:107–209. 10.1016/S0065-2296(08)00403-5

[39] Li HM, Rotter D, Bonos SA, Meyer WA, Belanger FC. Identification of a gene in the process of being lost from the genus Agrostis. Plant Physiol. 2005;138(4):2386–2395. 10.1104/pp.105.063297 15995002 PMC1183424

[40] Esen A, Blanchard DJ. A specific β-glucosidase-aggregating factor is responsible for the β-glucosidase null phenotype in maize. Plant Physiol. 2000;122(2):563–572. 10.1104/pp.122.2.563 10677449 PMC58893

[41] Kittur FS, Yu HY, Bevan DR, Esen A. Deletion of the N-terminal dirigent domain in maize beta-glucosidase aggregating factor and its homolog sorghum lectin dramatically alters the sugar-specificities of their lectin domains. Plant Physiol Biochem. 2010;48(8):731–734. 10.1016/j.plaphy.2010.03.007 20462765

[42] Jiang JF, Han Y, Xing LJ, Xu YY, Xu ZH, Chong K. Cloning and expression of a novel cDNA encoding a mannose-specific jacalin-related lectin from *Oryza sativa*. Toxicon. 2006;47(1):133–139. 10.1016/j.toxicon.2005.10.010 16359716

[43] Weidenbach D, Esch L, Molecularler C, Hensel G, Kumlehn J, Hofle C, et al. Polarized defense against fungal pathogens is mediated by the jacalin-related lectin domain of modular poaceae-specific proteins. Mol Plant. 2016;9(4):514–527. 10.1016/j.molp.2015.12.009 26708413

[44] Subramanyam S, Smith DF, Clemens JC, Webb MA, Sardesai N, Williams CE. Functional characterization of HFR1, a high-mannose N-glycan-specific wheat lectin induced by *Hessian fly larvae*. Plant Physiol. 2008;147(3):1412–1426. 10.1104/pp.108.116145 18467454 PMC2442546

[45] Nobile PM, Bottcher A, Mayer JLS, Brito MS, Dos Anjos IA, Landell MGA, et al. Identification, classification and transcriptional profiles of dirigent domain-containing proteins in sugarcane. Mol Genet Genomics. 2017;292(6):1323–1340. 10.1007/s00438-017-1349-6 28699001

[46] Ma Q H. Monocot chimeric jacalins: a novel subfamily of plant lectins. Critical Reviews in Biotechnology. 2014;34(4):300–306. 10.3109/07388551.2013.793650 23886351

[47] Lannoo N, Van Damme EJM. Nucleocytoplasmic plant lectins. Biochim Biophys Acta. 2010;1800(2):190–201. 10.1016/j.bbagen.2009.07.021 19647040

[48] Khan A, Li RJ, Sun JT, Ma F, Zhang HX, Jin JH, et al. Genome-wide analysis of dirigent gene family in pepper (*Capsicum annuum L.*) and characterization of CaDIR7 in biotic and abiotic stresses. Sci Rep. 2018;8(1):329–337. 10.1038/s41598-017-18514-4 29615685 PMC5883049

[49] Cheng X, Su XQ, Muhammad A, Li ML, Zhang JY, Sun YM, et al. Molecular characterization, evolution, and expression profiling of the dirigent (DIR) family genes in chinese white pear (*Pyrus bretschneideri*). Front Genet. 2018;9:136. 10.3389/fgene.2018.00136 29713336 PMC5911567

[50] Pickel B, Pfannstiel J, Steudle A, Lehmann A, Gerken U, Pleiss J, et al. A model of dirigent proteins derived from structural and functional similarities with allene oxide cyclase and lipocalins. FEBS Journal. 2012;279(11):1980–1993. 10.1111/j.1742-4658.2012.08580.x 22443713

[51] Ma QH, Zhen WB, Liu YC. Jacalin domain in wheat jasmonate-regulated protein Ta-JA1 confers agglutinating activity and pathogen resistance. Biochimie. 2013;95(2):359–365. 10.1016/j.biochi.2012.10.014 23116711

[52] Lannoo N, Van Damme EJM. Lectin domains at the frontiers of plant defense. Front Plant Sci. 2014;5:397. 10.3389/fpls.2014.00397 25165467 PMC4131498

[53] Andrade LM, Peixoto-Junior RF, Ribeiro RV, Nobile PM, Brito MS, Marchiori PER, et al. Biomass accumulation and cell wall structure of rice plants overexpressing a dirigent-jacalin of sugarcane (ShDJ) under varying conditions of water availability. Front Plant Sci. 2019;10:65. 10.3389/fpls.2019.00065 30815002 PMC6381051

[54] Li F, Wu M, Liu HL, Gao YM, Xiang Y. Systematic identification and expression pattern analysis of the Aux/IAA and ARF gene families in moso bamboo (*Phyllostachys edulis*). Plant Physiol Biochem. 2018;130, 431–444. 10.1016/j.plaphy.2018.07.033 30077919

[55] Zhang YT, Tang DQ, Lin XC, Ding MQ, Tong ZK. Genome-wide identification of MADS-box family genes in moso bamboo (*Phyllostachys edulis*) and a functional analysis of *PeMADS5* in flowering. BMC Plant Biol. 2018;18(1):176. 10.1186/s12870-018-1394-2 30176795 PMC6122543

[56] Wu RH, Shi YR, Zhang Q, Zheng WQ, Chen SL, Du L, et al. Genome-wide identification and characterization of the UBP gene family in moso bamboo (*Phyllostachys edulis*). Int J Mol Sci. 2019;20(17):4309. 10.3390/ijms20174309 31484390 PMC6747111

[57] Yang KB, Li Y, Wang SN, Xu XR, Sun HY, Zhao HS, et al. Genome-wide identification and expression analysis of the MYB transcription factor in moso bamboo (*Phyllostachys edulis*). PeerJ. 2019;6:e6242. 10.7717/peerj.6242 30648007 PMC6331034

[58] Wang TT, Wang HY, Cai DW, Gao YB, Zhang HX, Wang YS, et al. Comprehensive profiling of rhizome-associated alternative splicing and alternative polyadenylation in moso bamboo (*Phyllostachys edulis*). The Plant Journal, 2017;91(4):684–699. 10.1111/tpj.13597 28493303

[59] Zhao HS, Gao ZM, Wang L, Wang JL, Wang SB, Fei BH, et al. Chromosome-level reference genome and alternative splicing atlas of moso bamboo (*Phyllostachys edulis*). Gigascience. 2018;7(10):giy115. 10.1093/gigascience/giy115 30202850 PMC6204424

[60] Eddy SR. Accelerated Profile HMM Searches. PLoS Comput Biol. 2011;7(10):e1002195. 10.1371/journal.pcbi.1002195 22039361 PMC3197634

[61] Cheng YQ, Jiang SQ, Zhang XZ, He HL, Liu JF. Whole-genome re-sequencing of corylus heterophylla blank-nut mutants reveals sequence variations in genes associated with embryo abortion. Front Plant Sci. 2019;10:1465. 10.3389/fpls.2019.01465 31798613 PMC6863972

[62] Wilkins MR, Gasteiger E, Bairoch A, Sanchez JC, Williams KL, Appel RD, Hochstrasser DF. Protein identification and analysis tools in the ExPASy server. Methods Mol Biol. 1999;112:531–552. 10.1385/1-59259-584-7:531 10027275

[63] Petersen TN, Brunak S, von Heijne G, Nielsen H. SignalP 4.0: discriminating signal peptides from transmembrane regions. Nat Methods. 2011;8(10):785–786. 10.1038/nmeth.1701 21959131

[64] Chen CJ, Chen H, Zhang Y, Thomas HR, Frank MH, He YH, et al. TBtools: An integrative toolkit developed for interactive analyses of big biological data. Molecular Plant. 2020;13. 10.1016/j.molp.2020.06.009 32585190

[65] Wang Y, Tang H, Debarry JD, Tan X, Li J, Wang X, et al. MCScanX: a toolkit for detection and evolutionary analysis of gene synteny and collinearity. Nucleic Acids Res. 2012;40(7):e49. 10.1093/nar/gkr1293 22217600 PMC3326336

[66] Kumar S, Stecher G, Tamura K. MEGA7: molecular evolutionary genetics analysis version 7.0 for bigger datasets. Mol Biol Evol. 2016;33(7):1870–1874. 10.1093/molbev/msw054 27004904 PMC8210823

[67] Bailey TL, Boden M, Buske FA, Frith M, Grant CE, Clementi L, et al. MEME SUITE: tools for motif discovery and searching. Nucleic Acids Res. 2009;37(suppl2):W202–W208. 10.1093/nar/gkp335 19458158 PMC2703892

[68] Liu WZ, Xie YB, Ma JY, Luo XT, Nie P, Zuo ZX, et al. IBS: an illustrator for the presentation and visualization of biological sequences. Bioinformatics. 2015;31(20):3359–3361. 10.1093/bioinformatics/btv362 26069263 PMC4595897

[69] Lescot M, Déhais P, Thijs G, Marchal K, Moreau Y, Van de Peer Y, et al. PlantCARE, a database of plant cis-acting regulatory elements and a portal to tools for in silico analysis of promoter sequences. Nucleic Acids Res. 2002;30(1):325–327. 10.1093/nar/30.1.325 11752327 PMC99092

[70] Wang YS, Gao YB, Zhang HX, Wang HH, Liu XQ, Xu X, et al. Genome-wide profiling of circular RNAs in the rapidly growing shoots of moso bamboo (*Phyllostachys edulis*). Plant Cell Physiol. 2019;60(6):1354–1373. 10.1093/pcp/pcz043 30835314

[71] Kim D, Pertea G, Trapnell C, Pimentel H, Kelley R, Salzberg SL. TopHat2: accurate alignment of transcriptomes in the presence of insertions, deletions and gene fusions. Genome Biol. 2013;14(4):R36. 10.1186/gb-2013-14-4-r36 23618408 PMC4053844

[72] Trapnell C, Roberts A, Goff L, Pertea G, Kim D, Kelley DR, et al. Differential gene and transcript expression analysis of RNA-seq experiments with TopHat and Cufflinks. Nat Protoc. 2014;9:562–578. 10.1038/nprot1014-2513a PMC3334321 22383036

[73] Külahoglu C, Bräutigam A. Quantitative transcriptome analysis using RNA-seq. Methods Mol Biol. 2014;1158:71–91. 10.1007/978-1-4939-0700-7_5 24792045

[74] Li F, Zhang HZ, Wang SX, Xiao WF, Ding C, Liu WQ, et al. Identification of topping responsive proteins in tobacco roots. Front Plant Sci. 2016;7:582. 10.3389/fpls.2016.00582 27200055 PMC4848317

[75] Tateno H, Winter HC, Petryniak J, Goldstein IJ. Purification, characterization, molecular cloning, and expression of novel members of jacalin-related lectins from rhizomes of the true fern *Phlebodium aureum (L)* J. Smith (*Polypodiaceae*). J Biol Chem. 2003;278(13):10891–10899. 10.1074/jbc.M211840200 12538584

[76] Ma QH, Tian B, Li YL. Overexpression of a wheat jasmonate-regulated lectin increases pathogen resistance. Biochimie. 2010;92(2):187–193. 10.1016/j.biochi.2009.11.008 19958808 PMC7117000

[77] Khan F, Khan RH, Sherwani A, Mohmood S, Azfer MA. Lectins as markers for blood grouping. Med Sci Monit. 2002;8(12):RA293–RA300. 12503049

[78] Bourne Y, Zamboni V, Barre A, Peumans WJ, Van Damme EJM, Rougé P. Helianthus tuberosus lectin reveals a widespread scaffold for mannose-binding lectins. Structure. 1999;7(12):1473–1482. 10.1016/s0969-2126(00)88338-0 10647178

[79] Ralph S, Park JY, Bohlmann J, Mansfield SD. Dirigent proteins in conifer defense: gene discovery, phylogeny, and differential wound- and insect-induced expression of a family of DIR and DIR-like genes in spruce (*Picea spp.*). Plant Mol Biol. 2006;60(1):21–40. 10.1007/s11103-005-2226-y 16463097

[80] Meagher JL, Winter HC, Ezell P, Goldstein IJ, Stuckey JA. Crystal structure of banana lectin reveals a novel second sugar binding site. Glycobiology. 2005;15(10):1033–1042. 10.1093/glycob/cwi088 15944373

[81] Jiang B, Wang XJ, Wang LL, Lv XM, Li DM, Liu CH, et al. Two-Step isolation, purification, and characterization of lectin from zihua snap bean (*Phaseolus vulgaris*) seeds. Polymers (Basel). 2019;11(5):785. 10.3390/polym11050785 31052517 PMC6571848

[82] Raval S, Gowda SB, Singh DD, Chandra NR. A database analysis of jacalin-like lectins: sequence-structure-function relationships. Glycobiology. 2004;14(12):1247–1263. 10.1093/glycob/cwh140 15329359

[83] Chang WC, Liu KL, Hsu FC, Jeng ST, Cheng YS. Ipomoelin, a Jacalin-Related lectin with a compact tetrameric association and versatile carbohydrate binding properties regulated by its N terminus. PLoS ONE. 2012;7(10):e40618. 10.1371/journal.pone.0040618 22808208 PMC3394770

[84] Gallego del SF, Nagano C, Cavada BS, Calvete JJ. The first crystal structure of a Mimosoideae lectin reveals a novel quaternary arrangement of a widespread domain. J Mol Biol. 2005;353(3):574–583. 10.1016/j.jmb.2005.08.055 16185708

[85] Song M, Xu WQ, Xiang Y, Jia HY, Zhang LX, Ma ZQ. Association of jacalin-related lectins with wheat responses to stresses revealed by transcriptional profiling. Plant Mol Biol. 2013;84:95–110. 10.1007/s11103-013-0121-5 23959941

[86] Esch L, Schaffrath U. An update on jacalin-like lectins and their role in plant defense. Int J Mol Sci. 2017;18(7):1592. 10.3390/ijms18071592 28737678 PMC5536079

[87] Santner Aaron, Estelle Mark. Recent advances and emerging trends in plant hormone signalling. Nature. 2009;459(7250):1071–1078. 10.1038/nature08122 19553990

[88] Leng F, Cao J, Wang S, Jiang L, Li X, Sun C. Transcriptomic analyses of root restriction effects on phytohormone content and signal transduction during grape berry development and ripening. Int J Mol Sci. 2018;19(8):2300. 10.3390/ijms19082300 30082597 PMC6121528

[89] Chronopoulou L, Donati L, Bramosanti M, Rosciani R, Palocci C, Pasqua G, et al. Microfluidic synthesis of methyl jasmonate-loaded PLGA nanocarriers as a new strategy to improve natural defenses in *Vitis vinifera*. Sci Rep. 2019;9(1):18322. 10.1038/s41598-019-54852-1 31797901 PMC6892798

[90] Chen Y, Peumans WJ, Hause B, Bras J, Kumar M, Proost P, et al. Jasmonic acid methyl ester induces the synthesis of a cytoplasmic/nuclear chito-oligosaccharide binding lectin in tobacco leaves. FASEB J. 2002;16(8):905–907. 10.1096/fj.01-0598fje 12039875

