# Supplementary material for: Genome-wide identification and expression analysis of dirigent-jacalin genes from plant chimeric lectins in Moso bamboo (Phyllostachys edulis)
Source: PLoS One. 2021 Mar 16;16(3):e0248318. doi: 10.1371/journal.pone.0248318 (PMC7963094; doi:10.1371/journal.pone.0248318)
Supplement: S1 Table — (DOCX) [file pone.0248318.s001.docx]

**S1 Table.** **The DIR family renamed of Moso bamboo**

| DIR ID | ID Rename |
| --- | --- |
| PH02Gene10498.t1 | PeDIR04 |
| PH02Gene10505.t1 | PeDIR03 |
| PH02Gene10506.t1 | PeDIR02 |
| PH02Gene38476.t1 | PeDIR01 |
| PH02Gene21871.t1 | PeDIR06 |
| PH02Gene39117.t1 | PeDIR05 |
| PH02Gene41384.t1 | PeDIR07 |
| PH02Gene47479.t1 | PeDIR08 |
| PH02Gene17742.t1 | PeDIR09 |
| PH02Gene47979.t1 | PeDIR10 |
| PH02Gene46616.t1 | PeDIR11 |
| PH02Gene37326.t1 | PeDIR13 |
| PH02Gene37327.t1 | PeDIR14 |
| PH02Gene45653.t1 | PeDIR12 |
| PH02Gene15806.t1 | PeDIR17 |
| PH02Gene15808.t1 | PeDIR16 |
| PH02Gene15809.t1 | PeDIR15 |
| PH02Gene49830.t1 | PeDIR18 |
| PH02Gene09487.t1 | PeDIR24 |
| PH02Gene23504.t2 | PeDIR19 |
| PH02Gene27725.t1 | PeDIR20 |
| PH02Gene30332.t1 | PeDIR22 |
| PH02Gene30336.t1 | PeDIR23 |
| PH02Gene49479.t1 | PeDIR21 |
| PH02Gene44352.t1 | PeDIR25 |
| PH02Gene00136.t1 | PeDIR27 |
| PH02Gene03637.t1 | PeDIR28 |
| PH02Gene21283.t1 | PeDIR26 |
| PH02Gene49728.t1 | PeDIR29 |
| PH02Gene15063.t1 | PeDIR30 |
| PH02Gene15600.t1 | PeDIR31 |
| PH02Gene11037.t1 | PeDIR35 |
| PH02Gene18583.t1 | PeDIR34 |
| PH02Gene26302.t1 | PeDIR33 |
| PH02Gene31450.t1 | PeDIR32 |
| PH02Gene20575.t1 | PeDIR36 |
| PH02Gene36783.t1 | PeDIR38 |
| PH02Gene44204.t1 | PeDIR37 |
