# Supplementary material for: Genome-wide identification and expression analysis of dirigent-jacalin genes from plant chimeric lectins in Moso bamboo (Phyllostachys edulis)
Source: PLoS One. 2021 Mar 16;16(3):e0248318. doi: 10.1371/journal.pone.0248318 (PMC7963094; doi:10.1371/journal.pone.0248318)
Supplement: S2 Table — (DOCX) [file pone.0248318.s002.docx]

**S2 Table. The JRL family genes renamed of Moso bamboo**

| JRL ID | Rename |
| --- | --- |
| PH02Gene41919.t1 | PeJRL01 |
| PH02Gene45488.t1 | PeJRL02 |
| PH02Gene39024.t2 | PeJRL04 |
| PH02Gene39026.t1 | PeJRL03 |
| PH02Gene43609.t1 | PeJRL05 |
| PH02Gene08467.t1 | PeJRL06 |
| PH02Gene21121.t1 | PeJRL07 |
| PH02Gene03442.t1 | PeJRL08 |
| PH02Gene23335.t2 | PeJRL10 |
| PH02Gene25288.t1 | PeJRL09 |
| PH02Gene06516.t1 | PeJRL12 |
| PH02Gene31836.t1 | PeJRL11 |
| PH02Gene00698.t1 | PeJRL13 |
| PH02Gene25289.t1 | PeJRL15 |
| PH02Gene25354.t1 | PeJRL14 |
| PH02Gene07107.t1 | PeJRL16 |
| PH02Gene30596.t1 | PeJRL18 |
| PH02Gene30599.t2 | PeJRL17 |
| PH02Gene48979.t1 | PeJRL19 |
