# Supplementary material for: Genome-wide identification and expression analysis of dirigent-jacalin genes from plant chimeric lectins in Moso bamboo (Phyllostachys edulis)
Source: PLoS One. 2021 Mar 16;16(3):e0248318. doi: 10.1371/journal.pone.0248318 (PMC7963094; doi:10.1371/journal.pone.0248318)
Supplement: S3 Table — (DOCX) [file pone.0248318.s003.docx]

S3 Table. Conserved aa Sites of PeD-J family proteins

| Domain | Conserved aa sites | |
| --- | --- | --- |
| DIR domain | PeD-J01 | Ile113, Val114, Gly115, Gly116, Thy117, Gly118, Glu119, Phe120, Thr121, Met122, Ala123, Thr124, Gly125, Val126, Ile127, Ser128, Lys129, Lys130, Leu131 |
|  | PeD-J02 | Ile155, Val156, Gly157, Gly158, Thr159, Gly160, Lys161, Phe162, Ala163, Arg164, Ala165, Thr166, Gly167, Val168, Ile169, Ser170, Lys171, Lys172, Leu173 |
|  | PeD-J03 | Ile118, Val119, Gly120, Gly121, Thr122, Gly123, Gln124, Phe125, Ala126, Met127, Ala128, Thr129, Gly130, Val131, Ile132, Ser133, Lys134, Lys135, Thr136 |
|  | PeD-J04 | Ile117, Val118, Gly119, Gly120, Thy121, Gly122, Glu123, Phe124, Thr125, Met126, Ala127, Thr128, Gly129, Val130, Ile131, Ser132, Lys133, Lys134, Leu135 |
| JRL domain | PeD-J01 | Glyl63, Gly166, Gly170, Gly241, Tyr256, Asn257, Gly260, Pro261, Gly271, Phe276, Gly288, Phe289 |
|  | PeD-J02 | Gly202, Gly205, Gly209, Gly277, Thr295, Asn296, Gly302, Pro303, Gly305, Phe314, Gly325, Phe326 |
|  | PeD-J03 | Glyl68, Gly171, Gly175, Gly241, Thy259, Asn260, Gly265, Pro266, Gly268, Phe275, Gly287, Phe288 |
|  | PeD-J04 | Glyl67, Gly170, Gly174, Gly240, Tyr258, Asn259, Gly264, Pro265, Gly267, Phe274, Gly286, Phe287 |
