# Supplementary material for: Genome-wide identification and expression analysis of dirigent-jacalin genes from plant chimeric lectins in Moso bamboo (Phyllostachys edulis)
Source: PLoS One. 2021 Mar 16;16(3):e0248318. doi: 10.1371/journal.pone.0248318 (PMC7963094; doi:10.1371/journal.pone.0248318)
Supplement: S4 Table — (DOCX) [file pone.0248318.s004.docx]

**S4 Table. The primer of qRT-PCR of Moso bamboo and the primer of subcellular localization**

| ID | | | qRT-PCR primer |  |  |
| --- | --- | --- | --- | --- | --- |
| PeD-J02 | PeD-J2F | | TGAACGATAGCAGCTCGTGT |  |  |
|  | PeD-J2R | | GAACAGGGGGCAAATACAAA |  |  |
| PeD-J04 | PeD-J4F | | CTATTGTAGAAGGTGAATGG |  |  |
|  | PeD-J4R | | CGTGGATAGCAAGTTCTA |  |  |
| PeD-J03 | PeD-J3F | | ATATCTATGCCGGCAACTGG |  |  |
|  | PeD-J3R | | TGCGTCTTTTTGCTGATGAC |  |  |
| PeD-J01 | PeD-J1F | | GGTGAATGGGCTATTGTTGG |  |  |
|  | PeD-J1R | | CTTGAAATGTGCCTCCGTTT |  |  |
| NTB | NTBF | | TCTTGTTTGACACCGAAGAGGAG |  |  |
|  | NTBR | | AATAGCTGTCCCTGGAGGAGTTT |  |  |
|  | | |  | | |
| Name | | | Subcellular localization primer | | |
| 35S::PeD-J03-GFP-F  35S::PeD-J03-GFP-R | | | gagctcggtacccggggatcc ATGGCCAACCCCTCCAATTT  catgtcgactctagaggatccGAACGGATGAACGTA | | |
