# Supplementary material for: Genome-wide identification and expression analysis of dirigent-jacalin genes from plant chimeric lectins in Moso bamboo (Phyllostachys edulis)
Source: PLoS One. 2021 Mar 16;16(3):e0248318. doi: 10.1371/journal.pone.0248318 (PMC7963094; doi:10.1371/journal.pone.0248318)
Supplement: S5 Table — (DOCX) [file pone.0248318.s005.docx]

**S5 Table. The IDs and sequences of D-J proteins from rice, wheat, barley and maize using for phylogenetic tree**

| ID | ID Rename | Sequences |
| --- | --- | --- |
| HORVU1Hr1G002450.1 | HorD-J01 | MAATNPSYYQSGVCQDIKQKEHLFHLYMNQIFDGPGVTNGNQLSVINPGQPFGFGHTVANDWTIRDGPAANANLVARARGMHMGAGKVDENWLFCHNILFTDTRFKGSSLKVLGDFVGNNSEWAIVGGSGEFAYAQGVVVAKVIQTVPPTPGRTWELRISAFCLCIPKVIPVTKMGPWGGDGGTSFDITELPRSLQTVTIRCGDVINSVAFSYTDQAGQRKTVGPWGGNGALTATITLAPSEFIKQVLGTTGTVGGETVVTSLTLVSSITTYGPFGKANGSPFSSQVPDGNNIGGFYARAGESVNALGVYACPI |
| HORVU1Hr1G015190.1 | HorD-J02 | MADPSYYQRFPVTQQLQHTEHIFHLYVEQRSNQLPNGNQIPIAPTKPPSEPAGHFGFGVSCDWDVRDGPTPAAKTVARAQGLLLGTGQLTPFHYFLCQNIIFTDERFKGSSLKLLGSLPNDVNDEWAIIGGTGEFAFAQGSVKYKQVMVDPTGIVIRELNICVLCTNKPMKRIPYKDGPLGGKGGTICDITEAPQRLESVTIQSGDAIDSIMFSYTDQAGKKQMAGPWGGNDGLEQTILLAPTENVTKVFGTTGEFQGDTVVTSLTFVTNVATYGPFGKTKGTPFSIPKEDGDNVVGFFGR |
| HORVU2Hr1G013940.3 | HorD-J03 | PPNSPVTMANFQITPCATFVEVTELNFSNLYLFHTSLGSNQNQSVIIDSNATTGLGSTVVNNWSICDGPSPDATVVARAQGLHIYAGNWQNTFSITFEIERFKGSTLQVMGISVEEGEWAIVGGTGQFAMANGVIHKKFHEQRSDGNIIELTIHGFCPVLKSESLLTKLGPWGGNGGGDKDILEAVPRRMESITVSSGSIVDSIKFSYVDQTGQKHTAGPWGGSGGNQNTFMLGASEFVKEVSGTFGIYDKDRHNIITSLKFITNVKTYGPFGEAKGTPFTIPVQKNSSIVGFFGRSGIYLDALGVYVRPL |
| HORVU2Hr1G014020.2 | HorD-J04 | MATFQITPCAAFVEITELNFSNLYLFHTSLGSNQNQSVIIDSNATTGLGSTVVNNWSICDGPSPDATVVARAQGLHIYAGNWQNTFSITFEIERFKGSTLQVMGISVEEGEWAIVGGTGQFAMANGVIHKKLHEQRSDGNIIELTIHGFCPVLKSESLLTKLGPWGGNGGGDKDILEAVPRRLESITVSSGSIVDSIKFSYVDQTGQKHNAGPWGGSGGNQNTFVLGASEFMKEVSGTFGIYDKDRHNIITSLKFITNVKTYGPFGEAKGTPFTIPAQKNSSIVGFFGRSGIYLDALGVYVRPL |
| HORVU2Hr1G006090.1 | HorD-J05 | MAMDLPSNFELVDNSTLTEITKFTIKHVYLHHIYSGPNPNQLQLIAGTNGFGSTFVNSWEIYDGVGPKAHVIARADGLHMHSKSWHNSFSMLFEVERFKNSTLQVLGSSVEKEGEWAIVGGTGEFAMARGVIKIKVHEKIPGGEILELDILGYCTMPSRALAVTKSEAWGSNGNFVHDINEKPLRLRSVTVRTSDLFDGIKFTYIDQTGQTRTENLWGFSQDGSEHTVTLGPSEFLKEVSGVIDNSNEPNYFVRSIKLVTNQREFGPFGGAQGGTEFKSRVPEDSMIVAFHGSSGDNWPGHYRMHSIGVYSIPRNVLTT |
| HORVU3Hr1G082370.1 | HorD-J06 | MVNPSYFTSAPVTQEIHQNELNFHLYMYQWVEGSAQVNEKQVVNIAHPTGFGAIYANDWTMYDGPGPNANLVARAQGVHTGSDMNLGDSWLVCLNIVFVDQRFRGSTLKVVGNYGGLPVDEWAIVGGTGEFGFAHGVGTFRKYKEMGNGNMREFNIRVRCPTLQIPPVPTPPNDKEGPLGGKGGEAFDILGHPQRLESVTIRSGDIIDSLAFSYIDQAGKKQTAGPWGGNGGLEETITFAPTETLKKVFGVVGTIGGHTVVTSLTFVTNIKIYPTFGKGTGGTTFSIPEKNASVVGFFGRAGSYVDAIGVYIAN |
| HORVU3Hr1G098480.1 | HorD-J07 | PKNPFYRPTYLILPSYRPFHLSASMSNPTFYTNSAPHSIPPRQQQQEHQFKLYMSQIVEGTSGTNQLVVANNGRSDYFGITVAMDWVIRDDLGPKANIVAHAKGLHVGAGTPDWFTSFTIKFTHARFKDSTLMVQGIFGNSNIDVKGEWAIVGGTGQFAYAQGVVLYKQLSGLNGIVIRELEMHVLCPIFPKPSPVIKEGPWGGNGGTAYELPGWELPHRLQTVTVHGEGVIDSIAFTYVDEAGTKRSVGPFGGDGGVKNRPIILGPSETLREIYGTTGDYGGYTEVVTSLTVITSSNSVRTYGTQSNGKKTFRIAKDNHNIVGFYGRAGGFVDQIGAYLRPK |
| HORVU4Hr1G090780.3 | HorD-J08 | CKASQPLGCCLGQVVVYKCAHRSTHFASQEKLAEMAEPSYLQSVTLSQESQLKEVVLHLYLYQNSQAQPKGNQCQIVGSTRPHAFGATVVNDWTIHDGPSPTANIVARAQGLHLQAGMTAGNWFVCHNIVFTDDRFKGSTLNVLGNIEGQEGEWAIVGGTGEFAYAQGVVSYKPITSTTQGKVTRELHIRALCITLLKPPAPVVPTASTVAASSEMVAQAVVPPPAVPAPPVLPTRSEVVPTPVDFPMVLTKYGPWGPENGKYKDMQNELPQRLVRVTFFHGLYIYIMSFSYIDHSGVTKSCQLGGRPVSGSMPKEMNLEDGEFVKSVSGTTGLFDGDARLTSVSFVTNLRSYGPFGREGKHASFSTPELNNDSSIVGFFGWHVSNFAIDSTNYLSSLGVYVASPRAT |
| HORVU4Hr1G090720.1 | HorD-J09 | MCTPLHTFHQRIKTSGVRLLIAPMTDPSYVQSVTLSQKTQLKEFVLHLYLYQNREAQPKGNQFPIVGSTSPHAFGATVVNDWTIHDGPSPDAIIVARVQGLHLQAGMTASNWFICQNIVFTDDRLSGSTLKVMGNIQAGEGEWAIIGGTGEFAYAQGVASYKQMTSTPGGEVTRELQIRALCITLLKPPVPAVPTSTMPNPAEVLAPASSAPVPPARAVVVSTPDAVPMMYRKYGPWGSEKGKYFNMKTCDRITKVSVIYTSFIHTVQFSYIDRGINKSAQLGGQALPGSVVKEMSLEDGEYLRTFSGSTELIDGHARITSLSFGTNLRSYGRFGVEGRHSNFSITASNTNESIAYFFGWHESITGINEINYVNSVGVYLLGPSS |
| HORVU5Hr1G062640.1 | HorD-J10 | MANSSNFQTAPCDALMEYNELIFRDLYLHHPNSDKDKEKHTIIRVNGVSGFGATAVNNWEIYDGDRYDATVVAHGQGLHISAGNWHNTFSMVFDADRFMGSTLQVMGISIEDGEWAIVGGTGEFAMATGVIFKKLYKKSEHYSIIKLTIRAFCPLLKGSWSLPAKIGPWGGDGGTAQNMIKASRRLESITISSGDVVDSIAFSYIDEADQNCTTDRLGGPGGSSSTIQLAPSEFLTEVSGTISDGRCEVVESIQFVTNIQTYGPFGTQDGTPFTFSVPRYKKVVGFFGRGGLFLDAIGIYLQPV |
| HORVU7Hr1G121120.3 | HorD-J11 | MGDHPNFRCTPFDGGVENTEFEFSSLYLHHIYSGPCQTQSNIIGADATTGWGSTEVNNWTIYDGVGPCAKLVARAQGLHLYAGSWHNTFTLRFEIERFKKSTLQVMGASVDEEGEWSIVGGTGELAMARGVITKKLHARTDGGNIIQLTIHGFCRKLMPTLTKLGPWGGDRGFAVDSERPLKIESITILHEGIIGSIEYSYIDQNGNNRTAGPWGSRNPNRAEIGLGHGEMVTEVSGTFGTHSNGEAISTLKIVTNKKTYGPYGRIPGPKPFRAVAPHGKSIVGFFGRTDNIFLNALGVYIA |
| TraesCS7D02G434300.1 | TraD-J01 | MANFKVTGYAGALENTKLDFQSLYLHRINSGDKKNQYVVIDGFGTTDLGLTTINDWAIYDGVAPDAKLVARAKGIRVNAADWCNLFIIVFELDSFKGSTLSVMGATAEEQGEWAIVGGTGDFAMARGVIQRKIHQKVADGDVLELTIDAVCHRKGQPSPTPQPPAPSQPIQPPVQPRPAP  TPIKYGPWGGTGEKLYQFNPNMSVRLKSMTVFRHYAVNGLTFTYIGQDGQEHASELFGNSVLRGNKVDSQTFQLGPTEFLTGLSGTYGHESRSNNIAMYSLKLVTNLGNTHGPFGTPGIGTPFSFSVPQNNRIVSFFGGSTGYAINSIGIYTLDNSA |
| TraesCS2D02G060500.1 | TraD-J02 | MATPSSFKVTPFARLTENTEFDFRGLYLRNIVFGKNPNRALLVDLNASIGWGSTSVVNWTIYDGTCPDAQLVGRARGLQVDDDGWHNSLTLAFESGSYSKSLPMLTRAGPWGGAGDYTTYKDELEESWRIQSMTIVQDQGIIAMFECTYVDLSGKRRTTGAWGASHGHRVLNKIELGPQE  VLQALSGTYVDHNEETVIESLKFVTNEDEYGPYGRTTGIPFNYDVPEDRSIVGFFGRHGGKLIAIGVYMV |
| TraesCS3D02G437000.1 | TraD-J03 | MAAKDPSYLETTTLLSQEIQQKELRFKLYLFQHTQGEPNRNERAVSSLHAPHEFGSIVVHDWTIRDGPNLQDKIVARAQGLHLGAGMNETNWFTSFNIIFTDERFKGSTLQVLGTTSIRDGEWAITGGTGEFAFAQGVATHIKSKERGGAGRDWELRIRATCLTFPKPVLVTKIGPWGGH  GGKEFDIRESVPQHLESVTIRSGVAIDSIVFSYIDQAGKKQTLGPWGGDGELTDTITFAPLEIVKEVSGTSGTFGGDTVVTSLTFVTNVRTYGPFGKPNGTAFSVPLTDTNIVGFFVRAGRLVNAIGVYACPSVQNY |
| TraesCS7B02G479700.1 | TraD-J04 | MANPPNFHAASFDGAVENTEFNFRSLYLHHIFSGPNPTQAGIVSKDATTGWGSTVVNNWPIYDGVGHDAKLLARAQGLHINAGSWHNSFTIVFETERFKESTLQVMGTSVHEGQWSIVGGTADLAMARGVIEKKLHEKKNGGDIIELTIHGFCHMQIPTLVKSGPWGGNGGSAVDSEKPS  RIESITILYKGIIASFEYTYIDYYGNRRTSGPWGSENPNCAEIMLGPGEIVTAVSGTVIKHDTYVGTKVDVVQTLKFVTKKKTYGPYGNLDYNKELGTPFSAVAPDDKAIVGFFGRTDDKYLNEIGVYIA |
| TraesCS2B02G100800.1 | TraD-J05 | MANFQITPRAAFVESNELNFRSLYLFHTPLGSNQNQSGIIDSNVTTGLGATVVNNWPICDGPSPGATVIARAQGLHIYAGNWQNTFSITFEVERFKGSTLQVMGISVEEGEWAIVGGTGQFAMAIGVIYKKFHEQRSDGNIIELTVHGFCPMLKGSQSLRTKVGPWGGNGGSDKDIVEAPRRLESITVSSGTIIDSIKFSYVDQAGQKRTVGPWGGSGGKQNTFVLGTSEFVKEVSGTFGLYGRDNHNIITSLKFVTNVKTYGPFGQAKGTTFTIPVQKNSSIVGFFGRSGIYLDALGVYVHPL |
| TraesCS4A02G431400.1 | TraD-J06 | MACTPTDGPTMDVPMVHRSCPPRGGGVSTNFKVTCDFGGTVENAKVNVSKLYLRQIFAGCNANQSNVIQPNAATGLGKTVVNNWGIYDGPCSQAKLVAHGHGMHTLAGKWSNWFTLVFVAGRFKGSTLQVMGANDDDEENEWAIVGGTGEFSMARGVINKRVHSCIGNTITQELTIEFFCRMKEVVVCPPTQIAPPIEITAPTKIHAPIKQGPWGGMTGGSLHEMGGKSRRLESVTIYHHGAVEGLQFSYVDEDGQIHTTDTWGVNRGLFTNEIKFGPSEFVKQISGAGTLGSWLSQLKIVTNTNTYGPFGTIPSQAFSYTVPENATVVGFFAETLNVFITRIGVYTIPK |
| TraesCS1B02G016300.1 | TraD-J07 | MAATNPSYYQSGVCQDIKQKEHLFHLYMNQIFDGPGVTNANQVSVVNPPGQLFGFGHTIANDWTIRDGPAANANLVARARGMHMGAGKVDENWLFCHNILFTDTRFKGSSLKVLGDFVGNNSEWAIVGGSGEFAYAQGVVVAKVIQTVPPTPGRTWELRISAFCLCIPKAIPVTKMGPWG  GDGGTSFDITELPRSLQTVTIRCGDVINSVMFSYTDQTGQKKTAGPWGGDGALTATITLAPSEFIKQVLGTTGAVGGETIVTSLTLVSNVTTYGPFGKANGTPFSSQVPDGNNIGGFYARAGGSVNALGIYACPI |
| TraesCS1A02G010800.1 | TraD-J08 | MAATDPSYYQSGVCQDIKQKEHLFHLYMNQIFDGTPNANQQAIVKPGLPFGFGHTVANDWTIGDGPAADANLVARARGMHMGVGKVDENWLFCHNILFTDTRFKGSSLKVLGDFVSKEDSEWAIVGGTRKFAYAHGVVVAKILPNVPPAPARTWELRISAFCLCIPKVTPVTKMGLWGGN  GGISFDITEMPRSLQTVTIRCGDVINSVMFSYTDQAGHKKIAGPWGGDGALTVTITLAPSEFIRQVLGTTGTVGGETVVTSLSFVSSVTTYGPFGKANGTPFGSQVPDGNNIGGFYVRVGGFVNALGVYTCPS |
| TraesCS1B02G014200.1 | TraD-J09 | MAATDPSYYQSGVCQSITQKQHLFHLYINQIAEGTPNANQKVIVNPGLPMDFGVTVANDWTISDGPAANANPIARARGMHMGDGKADVNWLFCHNILFTDTRFKGSSLKVLGDFVANKDSEWAIVGGTGEFAYAQGVVVAKVIQNIQPTPGRTWELRISAFCLCIPNVTPVNKMGPWGADGGTSFDITELPRSLQTVTIRCGDVINSIGFSYTDQAGQKKIAGPWGGDGALSVAIKLAPSEFIKQVLGTTDAVGGVTVVTSLTLVSSVTTYGPFGKANGTPFSSQVTDSNTIGGFYARAGASVNSLGVYACPI |
| TraesCS4A02G190700.1 | TraD-J10 | MSPQPTLATTTTPATGPNASYFQAAPVSQETIQRTEVLLHLYAYQHAQGKPNANQTVIVDPKLPACFGALAANLVAHAQGLHIGAGMTKENWFICFNMVFVDQRFMGSSFKVMGDFRGNAGEWAIVGGTGEFAYAQGVITFNKTWSAQANVRELHVRALCLSFSKAPETPCSRTPRQSSV  TKIGPWGKISGEFLDVPTTPQRLKCVTIRHGVVIDSLAFSFVDQAGGQHNVGPWGGPCGHNKDTIELAPSEIVTEVSGTIGVFGEANVEYNAITSLTITTNVRTYGPFGEPQCTRFSVPVQDNSSIVGFFVCALKYVEALGVYVCPPVSN |
| TraesCS3D02G516700.1 | TraD-J11 | MDNFKITSYANALVENTKLDFQSLYLHRIRSGDKKNQYVVIDGVGATDICLTTINDWAIYDGAAVDAKLVAHAKGMHMNAAAADCCNLFIIVFELDSFKGSTLAVMGATTEEQGEWAIVGGTGQFAMARGVIQRKMHQKLADGQGDVLELTIEAFCRRKGQPEPPATTPQTPTTQPIQPPVQPPLATTQPIQPPVQPPPAPTKPIQPPTPTPIKNGPWGGTTSDTLHHFNPNMSKGLKSVTFIYYRAVNGLRFNYTGQDGYEESSELFGNNGQGPDAKRQTVHLGPKEFVTGLSGTYQHEGRSNNIAIYSLKLVTNLGKTHGPFGTASIGTGFSFLVPPNSRIVGFFGGSTGYAVNSIGAYTLENSA |
| TraesCS2A02G563700.1 | TraD-J12 | MASFQITPFAGLLENTEFNFHSLYLHNISSGPNPTRGAIINDNAINGWGQPFVVDWTIYDGTGHGAKLVGRAQGQQIYASKWSHSVTLEFTNGRFKGSTLQLMGLSATFEQPSEWSITGGTGDLAMARGIVKVKFHEMVEDGDTWELSFHGFCSMQSLPTLTKAGPWGGHGGSGTDSEQP  WRIESMTIVHEGTIAKYSCTYVDLSGKRRTTGSWGGGNGIPTKVQLGPREILKAVSGTHVCLNNGQTVIESLKFVTNEGTYGPFGHTTGTPFKANVPEDQSIVGFFGRADDMQLIAFGIYTV |
| TraesCS3B02G479000.1 | TraD-J13 | MAAKDLSYVKTTTPLDREIQQKEVSFHLYMFQTEETQRIVIKREENQRNSPSDFEAMAVQEWPIRDGPTFEANIVAHAVGLHFVVSRTEAKWFICFNIVFTDERLRPSNLKVLRTLVGMDGEWSIIGGTGKFAFVQGVATYKVIEVAEKYNVKELRIRALCLTFLPKQVLVTKIGPWGGN  GGKEFDIIESAPQHLESVTIRSGVAIDSIAFSYINQAGKKQTLGPWGGDGELTDTITFAPLEIVKEVSGTTGTFGGDTIVTSVTFVTNVRTYGPFGKPNGTAFSVPLTDTNVVGFFVRAGRPVNAIGVYARPSVQNY |
| TraesCS1B02G398600.1 | TraD-J14 | MAATDPSYYQSGVCQSITQKQHLFHLYMNQIAEGTPNANQKVIVNPGLPMDFGVTVANDWTISDGPAANANPIARARGMHMGDGKADVNWLFCHNILFTDTRFKGSSLKVLGDFVANKDSEWAIVGGTGEFAYAQGVVVAKVIQNIQPTPGRTWELRISAFCLCIPNMTPVNKMGPWGADGGTAFDITELPRSLQTMTIRCGDVINSIGFSYTDQAGQKKIAGPWGGDGALSVTIKLAPSEFMKQVLGTTDAVAGVTVVTSLTLVSSVMTYGPFGKANGTPFCSQVPDSNTIAGFYACWGGSVNALGVYACPI |
| TraesCSU02G178800.1 | TraD-J15 | MIQHKKLLLHLYAYQNVQKTPDANQAVIVESKHHECFGILAANDWTVYDGPGHNANLVAHAHGLHLGASMAKENWFICFNMVFVDQRNRFTGSSFKVMGDFQGIAHNGEWAIVGGTGEFAYAQGVIAFRKTQQSERNARIELHVRAMCLSFLRPLLSLGDGSAIAKIGPWGKISADLLDI  PTTPQRLERITIRHGVVIDSLAFSFIDKAGEPYNVGPWGGPRGDNKDTRSLRKSLGQLVSSQKIMSNIML |
| TraesCS1D02G007300.1 | TraD-J16 | MAATNPSYYQSGVCQDIKQKEHLFHLYMNQIFDGPGVTNANQVTVANPSGQLLGFGHTVANDWTIRDGPAADANLVARARGMHMGAGKVDENWLFCHNILFTDTRFKGSSLKVLGDFVGNNSEWAIVGGSGEFAYAQGVVVAKVIQTVPPTPGRTWELRISAFCLCIPKVTPVTKMGPWG  GDGGTSFDITELPRSLQTVTIRCGDVINSVMFSYTDQAGQKKTVGPWGGDGALTATITLAPSEFIKQVLGTTGTVGGETVVTSLTLVSSVTTYGPFGKANGTPFSSQVPDGNNVGGFYARAGRSVNALGVYACPI |
| GRMZM2G402417_P01 | ZmD-J01 | MAKLQVTPGAAFTECNELNFQGLYMYHTPLGPKANQAAILESKAKIGIGATVVNNWAVYDGPGPGAKLVGRAQGLHILAGNWVNSFTLVFEDERFSGSTLEVRGITVETGEWAVVGGTGQFAMANGIISKKLHEQRSDGNVIELSVHAFCPLLKGKRGGAVTKVGPWGGSGGSPMELTETETPMRLESITVSSGVAVNSISFSYVDSAGHKRSAGPWGGSGGQPDQVQLAESEVVTQVSGTYGTIDDDDRTVITSIKFVTNLDKTYGPFGAYGDGDDTSFTVPVQPGSGAIVGFFARVGGAGDYLDAIGVYVRPL |
| GRMZM2G046520_P01 | ZmD-J02 | MATPPNPPPPSTPIAAAAPLLPAEFTRFTFRALYVRRTGPSSREMIQPGRPYDELGRRLTSDYPVYDGRGSDAALVARMQGITVLVGNAHQLFTIVFETGRFKGSTLLTNGMVTEGSDEWAVYGGTGAFAMATGVIRRKNLLAGGGDSGGNSDELSVEVFCPVFGSPQQPPKDDDDGSAVTKIGLWGGPGGSAQDITTTEAPRRLNSVTIRAGIAVDSIEFTYTGKDRQRRTAGRWGGLGGNVRTIDLGDNEHIREVSGTYGTFEGATTLTSLRFITSTRAWGPWGTENGTRFCITAPIGSSVVGFYGRAGTRLVDAIGVYLRQL |
| GRMZM2G172204_P02 | ZmD-J03 | MASLQVTPTSAFTEWNELKFEGLYLFHTPLGSGANQARVIDNKAPIGIGATVVNNWTVYDGPGPNAKLVARAQGLHIQAGNWVNSFSLVFVDQRFSGSTLEVTGIVVESGEWAIVGGTGQFAMANGVIFKKFHEQRSEGNIIQLTIHAFCPVLGPRKRSAAKVGPWGGSGGSPVDITAEPQRLKSITVATGIAVTSIAFSYVDSAGQTQSAGRWGGSGGETEPVIQLGDSEVLTELSGTIGNVDGHTVITSIKFVTSLKTYGPFGAWGDGSDTPFAIPVQQGSAIVGFFARAGVYLDALGVYVRSL |
| GRMZM2G002630_P01 | ZmD-J04 | MENPPSILVTPMAPAMSAQFTRITFSNLFVRRAGPGSTELTVEGRPSDQLGRRNLTDSPVFDGRGPDASLVARVQGVATQMGDVRQLYTVVFQERPLKGSTLVTEGAMTEGSDEWAIYGGTGVFAMARGVIRRTFLADTSGGNSDELAVEVLCPVFRPAAFGSSSSQPAAKDISSTVVVTKVGVWGGEGGSAQDIATTEPPRRLQSLTVRAGVAVDSIEFTYSTDTGGQTRTAGRWGGLGGNVRKLDLGDAEYVKEVSGTYGAFEGATTLTSFRIVTSTARAWGPWGIESGTRFCITAPIGSSIVGFYGRATTRLVAAIGVYLRQL |
| GRMZM2G050412_P01 | ZmD-J05 | MENPAPSIVVAIAAPVAAEFVKLTFRALYMRRAGPGSREMTVPGRPSDQLGRRVVTDSPVYDGRGADARLVARIQGVTVLVGNADALFTMVFETDRLKGSTLVINGMVTDGSDEWAIYGGTGVFAMATGVMKRRNLAGGSNDGNSDELALEVFCPVFGASQQPISKQQESISSVTKIGLWGGQGGSAQDITAEQPPRRLHSITVRAGVAVDSIEFTYTDDAGQRRTAGRWGGLGGNVRTIDLGDAEDVREVSGTYGAFEGAITLTSLRLVTSSRTWGPWGVENGTRFSITAPNGSSIAGFYARAGTRLVDAIGVYIRPVVPGRPR |
| GRMZM2G025959_P01 | ZmD-J06 | MAENPAPSSPLVTSPDAAAPPLPAEFTKLTFRSLYIRRTGPGSREMTVDGRPSDQLGRRFVSDFPIYDGRGSGAHLVARLQGVTVQIGSSHQLVSIVFEAERLKGSTLLTNGVITDGSDEWAIYGGTGVFAMATGVIRRRFLAGSNDGNSDELTSIEVFCPAFGSAQQQPNKQDSVSVTKIGVWGGGGGSAQDITTTEPPQRLHSLSVRTGFAVDSIEFTYTDRGGQRRTAGRWGGLGGNLRTIDLGDAEVVREVSGTYGMFEGATTLTSIRILTSSRTWGPWGIEDGTRFCITAPIGSSIVGFYGRSTSRLVAAIGVYLRQQL |
| GRMZM2G163406_P01 | ZmD-J07 | MENPASVLVLPAQFTKLTFGNLYIRRAGPGSREITEEGRCDLDKRYVSDFPVYDGRGPDASLVARVQGITSEIGNAHQLFVVVFDTDRLKGSTLVTNGVITAGSDEWAIYGGTGVFAMARGVIRRRYLADRAGGNTDELNMDVFCRPFGSQSELQDKMQVQGQGSSVTKIGLWGGPGGSAQDITAERPPQRLHSVTVRAGVAVDSIEFTYTDSAGQRRAAGRWGGLGGNVRTIDLGDAEDVREVSGTYGAFEGATTLTSLRLVTSSRTWGPWGVENGTRFSVTAPIGSSIVGFYARAGTRLVDAIGVYLRQI |
| GRMZM2G112238_P03 | ZmD-J08 | MENPAPSIVVPPIAAPVSANFSRLAFRNLYIRRTGPDSREMVTVEGRRGSSDQTGDMRYVSDFPVYDGRGSDAVLVARVQGVTTTFGNSNQFFTVAFEAGSRLKGSTLLTEGVVTDGSDEWAIYGGTGEFAMARGVVKRRYLADRDGAGNTDELSMQVFCPVFGSSQQPNKQDSTISVTKIGLWGGEGGSAQDITTTEPPQRLHSLTVRASAAVDSIEFTYTDRGGQRRAAGRWGGLGGNLRTIDLGDAEDVREVSGTYGTFEGATTLTSFRILTSSRTWGPWGVENGTRFCITAPVGSSIVGFYGRATSRLVAALGVYLRRL |
| LOC_Os12g09700.1 | OsD-J01 | MSASKLQFTPCSTPIQGNEINFSKLYLHHTPAGPRPNQSGVTSTNKETGLGSLVVNNWQVYDGIGCDAKVVAHAQGLHVYAGNWHNSFTLVFEDERFKGSTLEVMGIVVEQGEWAIVGGTGQFAMANGVIFKKFHEQKKEGNIMELTIKGFCPVLKGSPSQGLVTKIGPWGGIDGGRAQD  ITATPKRLESITIHSGWTIDSISFIYFDQAGEKHRAGPWGGPGGDPCTIEFGSSEFLKEVSGTFGPYEGWKVIRSIKFVTNKKTYGPFGRQEGTPFSVPVQNNSTIVGFFGRSGKYLDTVGIYVHPR |
| LOC_Os12g09720.1 | OsD-J02 | MSASNNKLQFTPRSSLFQGNEINFSMLYLHHTPAGPRPDQSGLTGNNRETGLGPLVVNNWPVYDGIGRDAKVVARAQGLHIYAGNWHNSFSLVFKDERSGSTLEVMGIVVERGEWAIVGGTGQFAMANGVIFKKFHEQKQEGNIMELTIQGFCPVLKGSQLTLRDYIVNIMVLDINVLCP  LMKHLIFDSISFIYLDQAGQKHRAGPWGGPGGDPYMIEFGSSELLKEVSGTYGLYEGWKVIRSIKFVTNKKPYGPFGR |
| LOC_Os12g12720.1 | OsD-J03 | MAGINCSIVPCSALMEGKEFNFSNLYLHHTYGGPKPNQSTIINNNGSTGLGMTAVNNWAVYDGLGSDAKVVAHAHGLHIYAGDLVAIIPSAWYLRMKGEFIMASGVIYKKVHERRSEGNIIELTIHGFCPSLKGTKCLPTKVGPWGGNGGTPQDITETPKRLESITIRSGEVVDSISFSYFDQAGQKRVAGPWGGPGGNLNTIELSSSEFLKEVSGTFGTYYGSNVITFIKFVTNVKTYGPFGKQNGTPFISPCRTTAVL |
| LOC_Os12g14440.1 | OsD-J04 | MADPSKLQITPCGMLVQGNQINFTKLYLHHTPAGPEQNQSAVTSNDKKTGLGCIVVNNWSVYDGIGSDAKLVAYAKGLHVFAGAWHNSFSLVFEDERLKGSTLQVMGLIVEEGDWAIVGGTGQFAMATGVILKKMQEQKQYGNIIELTIHGFCPLLKGSQCPVTKIGPWGSSHEGTVQDI  TESPKRLESITLYHGWSVDSISFTYLDHAGEKHKAGPWGGPGGDPIMIEFGSSEFLKEVSGTFGPYEGSTVITSINFITNKQTYGPFGRQEGTPFSVPAQNNSSIVGFFGRSGKYINAVGVYVQPI |
| PH02Gene23777.t1 | PeD-J01 | MANPCNFQITPCVALVENNEINFRSLFLYHTTPPVNHIWAHNNREMKQGTAAALINWPVYDGPGLNATLVARAQGLHIYAGNNTLSLVFEVGRFKGSTLQVMGISVTEGEWAIVGGTGEFTMATGVISKKLHEQRKDGYTIELAIRALCPLPKGSRSLLTKIGPWGGNGGTFQDIRGAPRRLESITIYRIKDKVVNSIAFSYIDHDGEKRTAGPWGGSGGERYKIKLDSSEFVKEVSGTFGTYEGSTVITSLKFVTNVKEYRHLGDYLNSGKGTLFTVPVQDNGSVVGFFGRSGKYFLHAVGVYVHPF |
| PH02Gene23778.t1 | PeD-J02 | MISRTCSACSSPKFALRTRDSFRPFPSGLSSWLLNYAALAFYSPPSVSRFSISLVSHSISATAMANPSNFQITPCVALVENNEINFRSLYFRATELGTPTGHINWTVYDGPDEESVVARAQGVHNTFSLVFEVERFKGSTLQVMGLFVVQ  GEWAIVGGTGKFARATGVISKKLHEKKSKIELTIHAICPLPKGSLSLVPKIGPFGGDKGTKHDIAEAPRRLESITVYSVEKKVVNSIAFSYIDHTGRKHKTGSWGGSGGERHQIELDPSKFVKEVSGTFGTYKGYTVITSLMFVTNDNKTRGPYGDSSAKETRFSVPVQDSSVVGFFGRSGTDFLHAVGVYVHPF |
| PH02Gene03445.t1 | PeD-J03 | MANPSNFQITPCVALVESNEINFRSLYLYHTWVSPNLNQAEIIEKNSSTGMGMTSVNNWTVYDGPGPNATLVARGQGLHIYAGNWHNTFSLVFEVERFKGSTLQVMGITVDQEGEWAIVGGTGQFAMATGVISKKTHERRNDGNIIELAIHAFCPLPKASRSLLTKIGPWGGNGGTAQDITEAPRRLESITVCSGEIVDSIVFSYIDHTGQKRTAGRLGGSGGHPNTIQLASSEFVKEVSGTICTCEDSNVITSLKFVTNVKTYGPFGQGNGTPFTVPVQDNSSVVGFFGRGETYLDAIGVYVHPF |
| PH02Gene07832.t1 | PeD-J04 | MANPSNFQITPCVALVENNEINFRSLYLYHTYLGPKLNQSGIVNANSATGLGATVVNNWTVYDGTDPNATLVARAQGLHINAGNWHNTFSLVFEVERFKGSTLQVMGISIVEGEWAIVGGTGEFTMATGVISKKLHKNTKGEDIIELAIHAFCPLPKGSRSLLTKIGPWGGNGGTAQDITEVPRRLESITVCSGTVVDSIAFSYTDHTGQKRTAGRLGGSGGNPNTIQLASSEFVKEVSGTFATFQGCNV  ITSLKFVTNVKTYGPFGQGNGTPFTVPVQDNSSVVGFFGRGGTYLDAVGVYVLPF |
