# Supplementary material for: Genome-wide identification and expression analysis of dirigent-jacalin genes from plant chimeric lectins in Moso bamboo (Phyllostachys edulis)
Source: PLoS One. 2021 Mar 16;16(3):e0248318. doi: 10.1371/journal.pone.0248318 (PMC7963094; doi:10.1371/journal.pone.0248318)
Supplement: S6 Table — (DOCX) [file pone.0248318.s006.docx]

**S6 Table. The accession numbers of transcriptome data**

| Number | Accession |
| --- | --- |
| SRR6171235 | GSM2810849 |
| SRR6171236 | GSM2810850 |
| SRR6171237 | GSM2810851 |
| SRR6171238 | GSM2810852 |
| SRR6171239 | GSM2810853 |
| SRR6171240 | GSM2810854 |
| SRR6171241 | GSM2810855 |
| SRR6171242 | GSM2810856 |
| SRR6171243 | GSM2810857 |
| SRR6131113 | GSM2803908 |
| SRR6131114 | GSM2803909 |
| SRR6131115 | GSM2803910 |
| SRR6131116 | GSM2803911 |
| SRR6131117 | GSM2803912 |
| SRR6131118 | GSM2803913 |
| SRR5710697 | GSM2673736 |
| SRR5710698 | GSM2673737 |
| SRR5710699 | GSM2673738 |
| SRR5710700 | GSM2673739 |
| SRR5710701 | GSM2673740 |
| SRR5710702 | GSM2673741 |
| ERR105067 | ERX082501 |
| ERR105069 | ERX082503 |
| ERR105073 | ERX082507 |
| ERR105075 | ERX082509 |
