# Supplementary material for: Genome-wide identification and expression analysis of dirigent-jacalin genes from plant chimeric lectins in Moso bamboo (Phyllostachys edulis)
Source: PLoS One. 2021 Mar 16;16(3):e0248318. doi: 10.1371/journal.pone.0248318 (PMC7963094; doi:10.1371/journal.pone.0248318)
Supplement: S1 Fig — The blue circle indicates DIR genes and the orange circle indicates JRL genes. The 4 PeD-J genes in the middle are crossovers. (DOCX) [file pone.0248318.s007.docx]

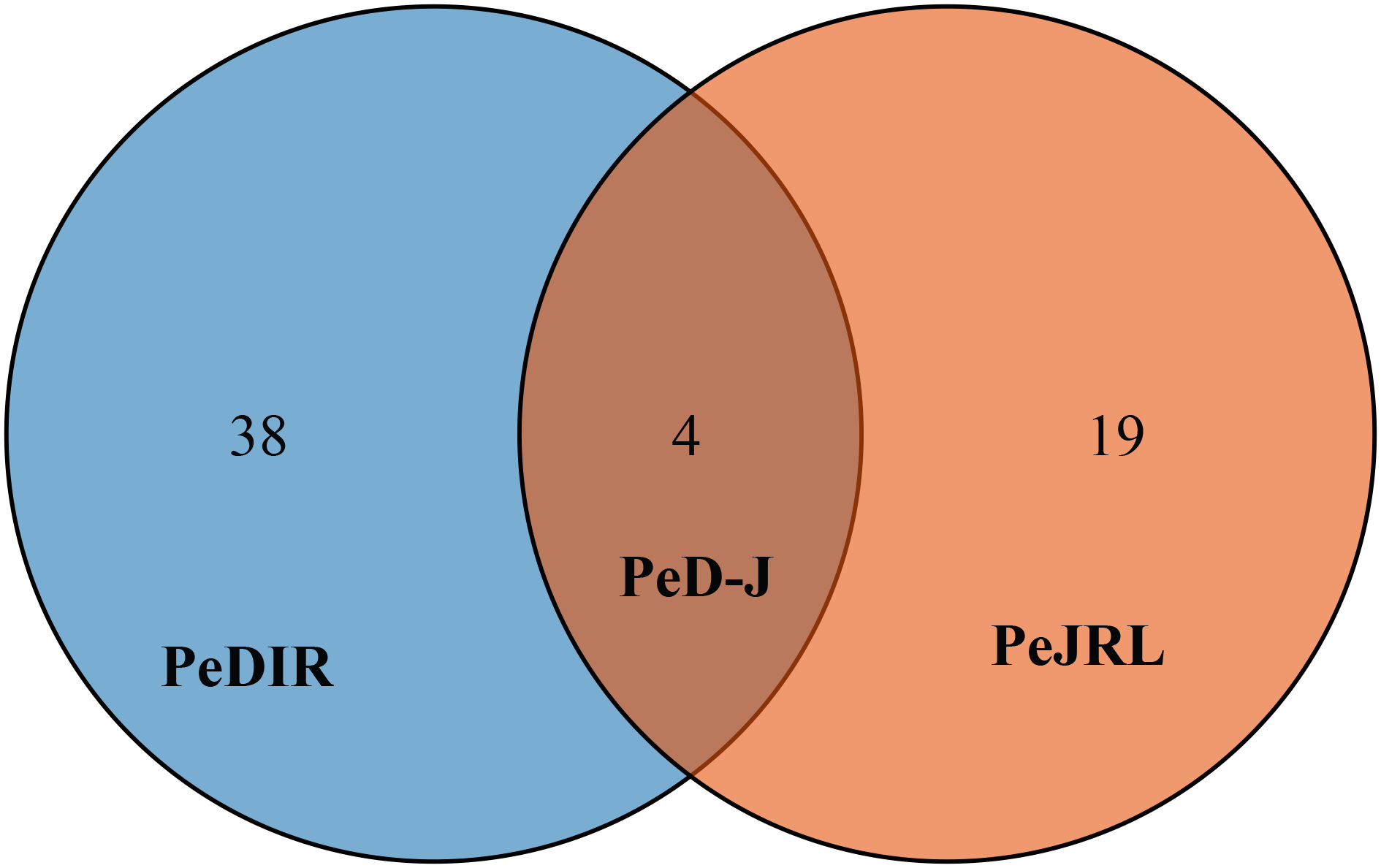


S1 Fig. The venn graph of PeDIR and PeJRL. The blue circle indicates DIR genes and the orange circle indicates JRL genes. The 4 *PeD-J* genes in the middle are crossovers.
