# Supplementary material for: Genome-wide identification and expression analysis of dirigent-jacalin genes from plant chimeric lectins in Moso bamboo (Phyllostachys edulis)
Source: PLoS One. 2021 Mar 16;16(3):e0248318. doi: 10.1371/journal.pone.0248318 (PMC7963094; doi:10.1371/journal.pone.0248318)
Supplement: S2 Fig — The blue amino acids are conserved amino acid residues and the black amino acids are binding substrates. (DOCX) [file pone.0248318.s008.docx]

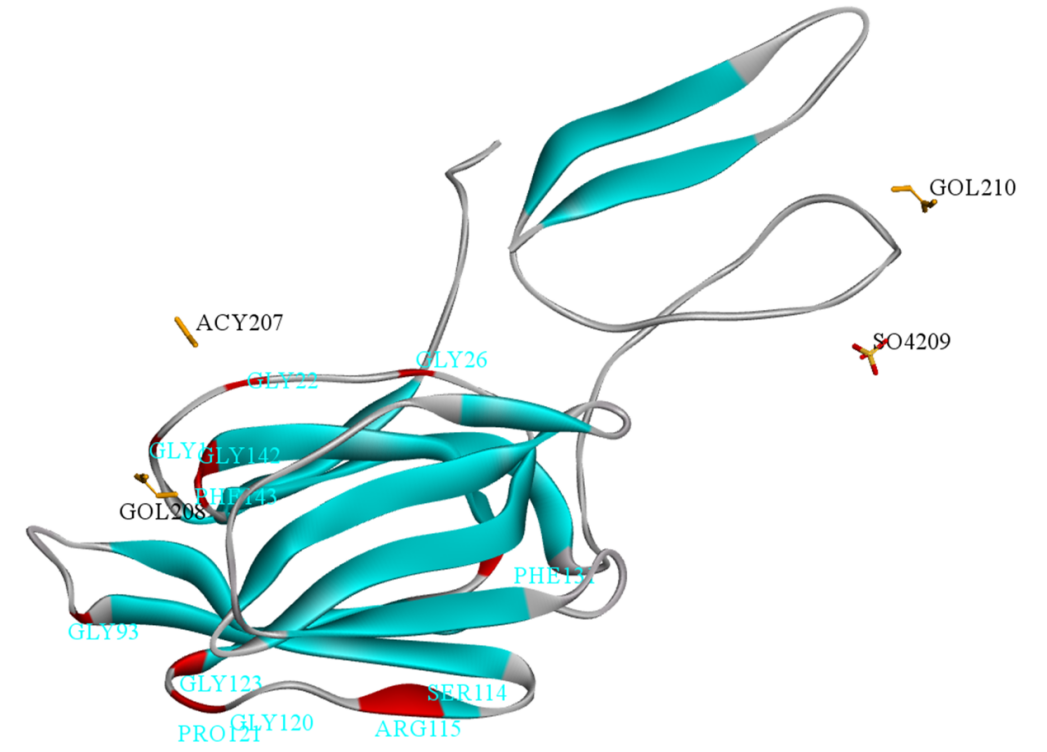


PeJRL12

S2 Fig. **Protein structures based on homologous modeling of PeJRL12.** The blue amino acids are conserved amino acid residues and the black amino acids are binding substrates.
