# Supplementary material for: Genome-wide identification and expression analysis of dirigent-jacalin genes from plant chimeric lectins in Moso bamboo (Phyllostachys edulis)
Source: PLoS One. 2021 Mar 16;16(3):e0248318. doi: 10.1371/journal.pone.0248318 (PMC7963094; doi:10.1371/journal.pone.0248318)
Supplement: S3 Fig — The light purple amino acids are conserved amino acid residues in DIR domain. (DOCX) [file pone.0248318.s009.docx]

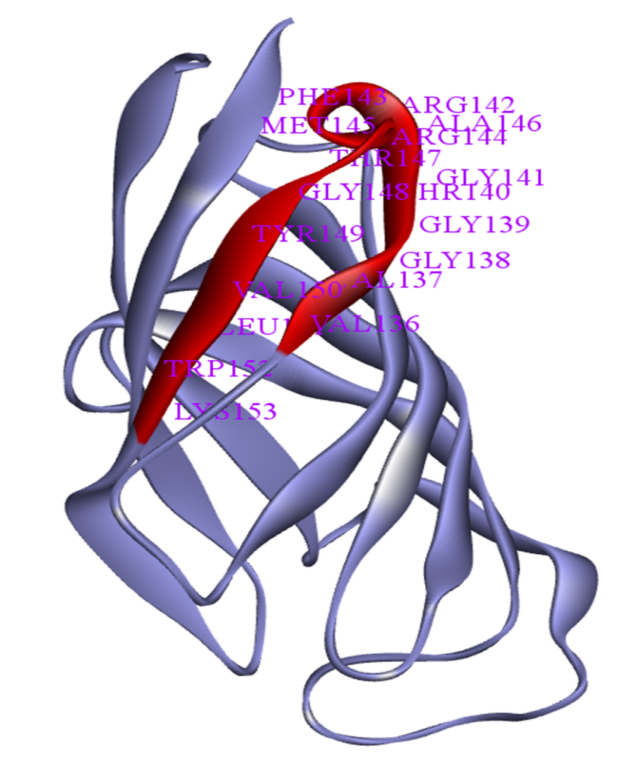


PeDIR08

S3 Fig. **Protein structures based on homologous modeling of PeDIR08.** The light purple amino acids are conserved amino acid residues in DIR domain.
