# Supplementary material for: Genome-wide identification and expression analysis of dirigent-jacalin genes from plant chimeric lectins in Moso bamboo (Phyllostachys edulis)
Source: PLoS One. 2021 Mar 16;16(3):e0248318. doi: 10.1371/journal.pone.0248318 (PMC7963094; doi:10.1371/journal.pone.0248318)
Supplement: S4 Fig — The yellow chain represents the conserved amino acid residues of the dirigent domain region. The red chain represents the conserved amino acid residues involved in β-prismatic folding of the jacalin domain region. (DOCX) [file pone.0248318.s010.docx]

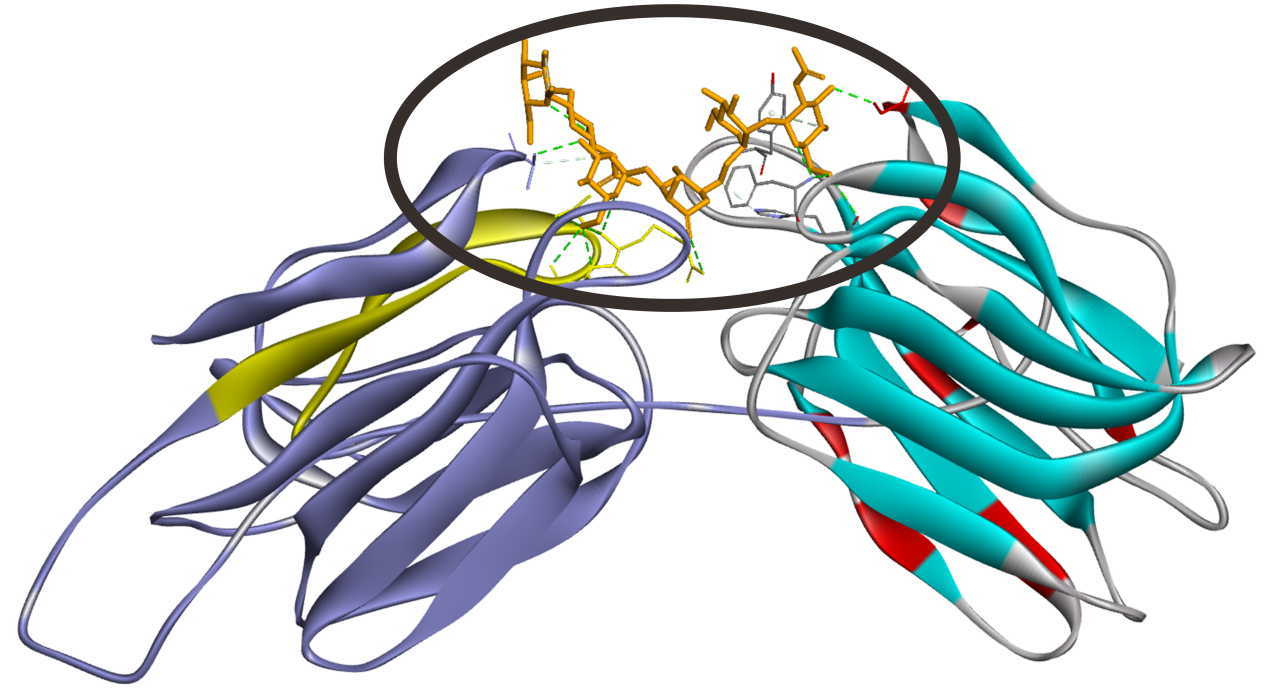


S4 Fig. **Protein structures based on homologous modeling of PeD-J02.** The yellow chain represents the conserved amino acid residues of the dirigent domain region. The red chain represents the conserved amino acid residues involved in β-prismatic folding of the jacalin domain region.
