# Supplementary material for: Genome-wide identification and expression analysis of dirigent-jacalin genes from plant chimeric lectins in Moso bamboo (Phyllostachys edulis)
Source: PLoS One. 2021 Mar 16;16(3):e0248318. doi: 10.1371/journal.pone.0248318 (PMC7963094; doi:10.1371/journal.pone.0248318)
Supplement: S5 Fig — The diagram shows the important covalent bonds to which the substrate is bound as well as the important amino acid residues. (DOCX) [file pone.0248318.s011.docx]

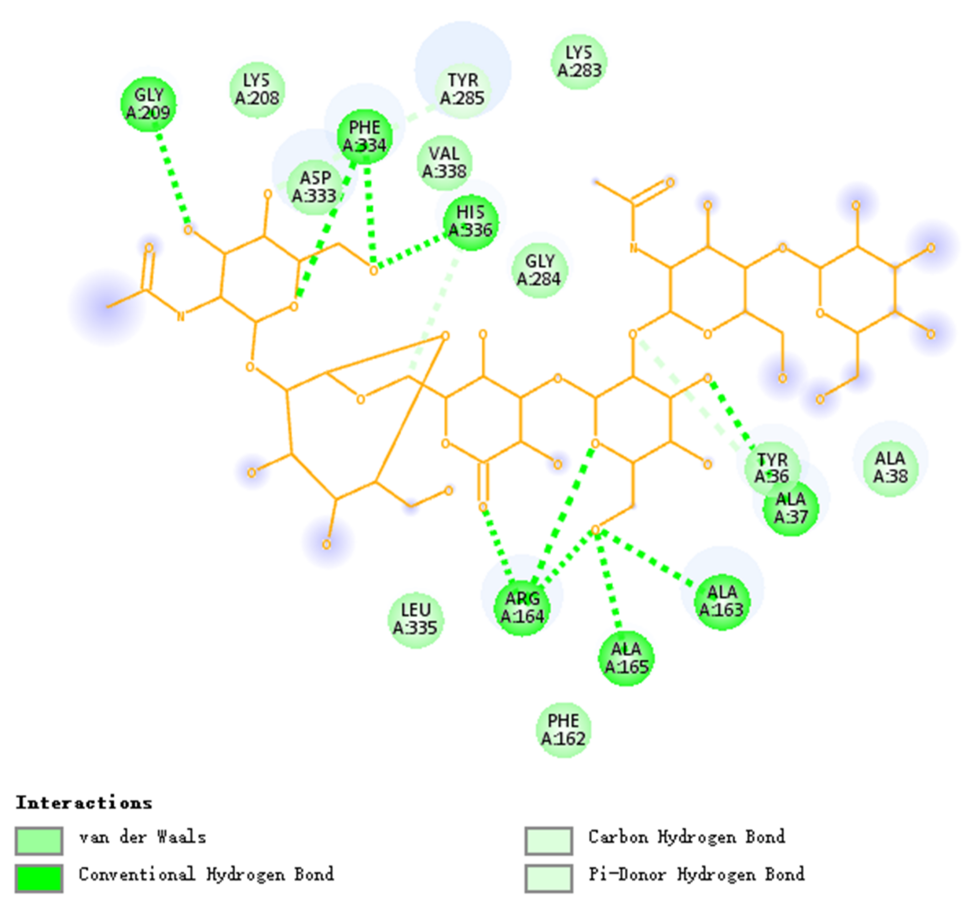


S5 Fig. The 2D diagram of the binding site of PeD-J02. The diagram shows the important covalent bonds to which the substrate is bound as well as the important amino acid residues.
