# Supplementary material for: Genome-wide identification and expression analysis of dirigent-jacalin genes from plant chimeric lectins in Moso bamboo (Phyllostachys edulis)
Source: PLoS One. 2021 Mar 16;16(3):e0248318. doi: 10.1371/journal.pone.0248318 (PMC7963094; doi:10.1371/journal.pone.0248318)
Supplement: S6 Fig — The yellow chain represents the conserved amino acid residues of the dirigent domain region. The red chain represents the conserved amino acid residues involved in β-prismatic folding of the jacalin domain region. The blue amino acid residues (GLY221, GLY222, GLY291, GLY292, THR293, TYR294, LEU295 and ASP296) are the second sugar binding site. NAG306 is the binding substrate. (DOCX) [file pone.0248318.s012.docx]

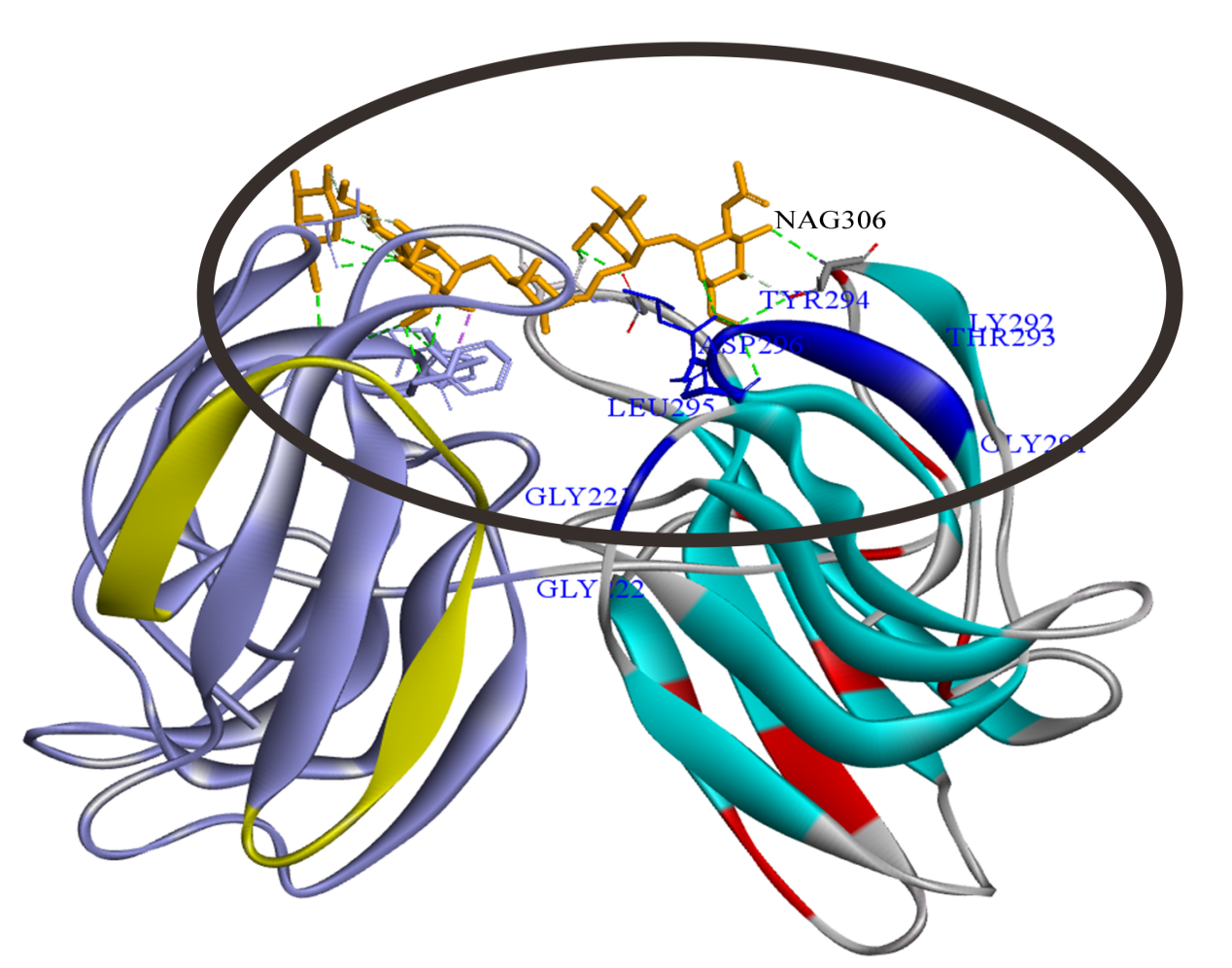


S6 Fig. **Protein structures based on homologous modeling of PeD-J04.** The yellow chain represents the conserved amino acid residues of the dirigent domain region. The red chain represents the conserved amino acid residues involved in β-prismatic folding of the jacalin domain region. The blue amino acid residues (GLY221, GLY222,GLY291, GLY292, THR293, TYR294, LEU295 and ASP296) are the second sugar binding site. NAG306 is the binding substrate.
